# Tumor-targeted superantigens produce curative tumor immunity with induction of memory and demonstrated antigen spreading

**DOI:** 10.1186/s12967-023-04064-z

**Published:** 2023-03-26

**Authors:** Meir Azulay, Michal Shahar, Eitan Shany, Eti Elbaz, Sveta Lifshits, Marie Törngren, Adam Friedmann, Robert Kramer, Gunnar Hedlund

**Affiliations:** 1 NeoTX Therapeutics LTD, Rehovot, Israel; 2 Active Biotech AB, Lund, Sweden; 3 Department of Genetics, The Hebrew University, Jerusalem, Israel; 4 ImmunoPoint Consulting AB, Lund, Sweden

**Keywords:** Naptumomab estafenatox, Immune checkpoint inhibitors, Programmed cell death 1 receptor, T-cell receptors, Memory T lymphocytes, Immunotherapy, Superantigens, Tumor infiltrating lymphocyte, Antigen-presenting cells

## Abstract

**Background:**

Despite remarkable progress, the immunotherapies currently used in the clinic, such as immune checkpoint blockade (ICB) therapy, still have limited efficacy against many types of solid tumors. One major barrier to effective treatment is the lack of a durable long-term response. Tumor-targeted superantigen (TTS) therapy may overcome this barrier to enhance therapeutic efficacy. TTS proteins, such as the clinical-stage molecule naptumomab estafenatox (NAP), increase tumor recognition and killing by both coating tumor cells with bacterial-derived superantigens (SAgs) and selectively expanding T-cell lineages that can recognize them. The present study investigated the efficacy and mechanism of action of repeated TTS (C215Fab-SEA) treatments leading to a long-term antitumor immune response as monotherapy or in combination with PD-1/PD-L1 inhibitors in murine tumor models.

**Methods:**

We used syngeneic murine tumor models expressing the human EpCAM target (C215 antigen) to assess the efficacy and mechanism of action of repeated treatment with TTS C215Fab-SEA alone or with anti-PD-1/PD-L1 monoclonal antibodies. Tumor draining lymph nodes (TDLNs) and tumor tissues were processed and analyzed by immunophenotyping and immunohistochemistry. Isolated RNA from tumors was used to analyze gene expression and the TCR repertoire. Tumor rechallenge and T-cell transfer studies were conducted to test the long-term antitumor memory response.

**Results:**

TTS therapy inhibited tumor growth and achieved complete tumor rejection, leading to a T-cell-dependent long-term memory response against the tumor. The antitumor effect was derived from inflammatory responses converting the immunosuppressive TME into a proinflammatory state with an increase in T-cell infiltration, activation and high T-cell diversity. The combination of TTS with ICB therapy was significantly more effective than the monotherapies and resulted in higher tumor-free rates.

**Conclusions:**

These new results indicate that TTSs not only can turn a “cold” tumor into a “hot” tumor but also can enable epitope spreading and memory response, which makes TTSs ideal candidates for combination with ICB agents and other anticancer agents.

**Supplementary Information:**

The online version contains supplementary material available at 10.1186/s12967-023-04064-z.

## Background

Despite the tremendous development of new immuno-oncology treatments using vaccines, adoptive cell transfer, and immune checkpoint blockades (ICBs), many patients with solid cancers do not benefit from these therapies. This can be due to low immunogenicity and immune suppression that results in limited tumor-associated T-cell activation [1, 2]. Therapeutic cancer vaccines rely on T-cell activation but have had limited success in the clinical setting to date [3]. Other efforts to improve T-cell activation have focused on the T-cell receptor (TCR) complex, which consists of several proteins. These have been successfully engineered to create transferable CAR-T cells [4]. Another approach has been to develop targeted therapies that activate and redirect T cells to recognize specific tumor-associated antigens (TAAs).

Most targeted T-cell therapies engage T cells via the CD3 [5] component of the TCR complex, and the clinical activity of this approach has been demonstrated against various hematological malignancies expressing a range of tumor-specific antigens. Unfortunately, this therapy has had limited success against solid tumors. One explanation for the lack of efficacy in solid tumors is artificial T-cell activation mediated via CD3 that is independent of the CD28 costimulatory signal [6]. Tumor-targeted superantigens (TTSs) are fusion proteins that consist of genetically engineered bacterial superantigens linked to fragment antigen binding (Fab) moieties directed to tumor-associated antigens [7]. In contrast to the pan-T-cell activation approach using CD3-targeted constructs, the SAg moiety of the TTS binds selectively to ~ 4% of T cells via distinct variable regions of the TCR β-chain and to the class II major histocompatibility complex (MHC) expressed on professional antigen-presenting cells (APCs), resulting in T-cell activation that is supported by costimulation, such as the CD28–CD80/CD86 interaction [7–9]. Treatment with TTSs leads to the expansion and differentiation of specific Vβ subsets of T cells in lymph nodes (Vβ3 [10] in mice and TRBV7-9 [8] in humans for SEA and SEA/E-120-containing TTSs, respectively), and these TTS-reactive T cells then infiltrate tumor tissues that express the tumor antigen recognized by the Fab moiety of the TTS construct. In the tumor, Vβ T cells are subsequently activated by TCR binding to the superantigen tethered to the tumor via the Fab bound to the appropriate tumor antigen, resulting in the production of cytokines and direct tumor cell killing [7–9, 11, 12]. The antitumor activity of TTSs has been confirmed in murine tumor models and was shown to be T-cell dependent. TTS (C215Fab-SEA) treatment induced massive tumor infiltration of CD4 + and CD8 + T cells, while only scattered T cells were observed in untreated tumors [13]. In addition, there is also clinical evidence of selective T-cell activation and T-cell tumor infiltration in solid tumors following TTS (NAP) treatment [14, 15].

Here, we present new evidence that tumor-selective T-cell activation by TTS produces broad antitumor immune memory responses as monotherapy and in combination with PD-1/PD-L1 treatment that lead to the eradication of tumors, with a long-lasting antitumor response, in mice. These studies have led to the initiation of a phase 1 study of the 5T4-targeted TTS NAP in combination with durvalumab in solid tumors [NCT03983954]. NAP is currently also being evaluated in a phase 2 study in combination with docetaxel in the treatment of NSCLC [NCT04880863].

## Methods

### Cell lines

The B16F10-hEpCAM [16], MC38 parental and MC38 hEpCAM [17] cell lines were grown in RPMI 1640 (Gibco Paisley, Scotland, UK) supplemented with 10% fetal bovine serum (Gibco, Grand Island, New York), 1% sodium pyruvate (Gibco), 1% pen/strep (Gibco), 1% Glutamax (Gibco), 1% nonessential amino acids (Gibco), 0.1% beta-mercaptoethanol (Gibco), and 0.5 or 1 mg/ml G-418 solution (Roche, Rotkreuz, Switzerland) and were maintained at 37 °C and 5% CO_2_ for at least three passages.

### Animals and treatment

For the murine studies, recombinant C215Fab-SEA was expressed in *Escherichia coli* and purified as previously described [16]. Since BALB/c mice show clonal deletion of most Vβ3 + immature thymocytes by mammary tumor virus superantigens [18], the in vivo efficacy of C215Fab-SEA was restricted to model tumors of the C57BL/6 syngeneic strain background.

Female C57BL/6 mice (Taconic, M & B A/S, Denmark) 8–12 weeks of age were used for the B16F10-hEpCAM studies. The studies were approved by the local animal ethical committee (Lund, Sweden). Mice were injected intravenously (i.v.) with 125,000 B16F10 hEpCAM cells in the tail vein. On Day 3 postinoculation, C215Fab-SEA or vehicle (PBS with 1% C57BL/6 serum) was i.v. injected (200 µl; 0.5 µg/mouse; 0.025 mg/kg) weekly for four consecutive days (a single treatment cycle) for a total of two treatment cycles (Cycle 1: Days 3–6; Cycle 2: Days 24–27). For ICB combination therapy, anti-mouse PD-1 (Rat IgG2a; Bioxcell, New Haven, Connecticut, United States) monoclonal antibody (mAb) or Rat IgG2a isotype (Bioxcell) were intraperitoneally (i.p.) injected biweekly (200 µl; 200 µg/mouse; 10 mg/kg) starting on Day 3 postinoculation for 3 weeks. The survival of mice was monitored up to 90 days after tumor inoculation. For immunohistochemistry (IHC) analysis, mice were i.v. injected with 175,000 B16F10 hEpCAM cells in the tail vein and randomized to a single treatment cycle of C215Fab-SEA (200 µl; 0.5 µg/mouse; 0.025 mg/kg) on Days 5–8 postinoculation with or without anti-mouse PD-1 (Rat IgG2a; Bioxcell) mAb or Rat IgG2a isotype (Bioxcell) i.p. injected biweekly (200 µl; 200 µg/mouse; 10 mg/kg) starting on Day 5 postinoculation for a total of 4 injections. On Day 21 postinoculation, the mice were sacrificed, and the lungs were dissected and snap frozen for IHC analysis.

MC38-hEpCAM tumor studies were carried out in accordance with the Guide for the Care and Use of Laboratory Animals of Tel Aviv University (TAU; Tel Aviv, Israel). All protocols were approved by the TAU Institutional Animal Care and Use Committee (IACUC). A total of 500,000 MC38 hEpCAM cells were subcutaneously (s.c.) injected into the right flank of 7- to 8-week-old C57BL/6 J female mice (Envigo RMS; Jerusalem, Israel). Five to seven days postinoculation, the tumors were measured, and mice were randomized into four groups of 10–15 mice/group (mean tumor volume of ≈60 mm^3^). C215Fab-SEA or vehicle (PBS with 1% C57BL/6 serum) was i.v. or i.p. injected (200 µl; 15 or 20 µg/mouse; 0.75 or 1 mg/kg) weekly for four consecutive days (a single treatment cycle) for a total of three to four treatment cycles (C1-C4). For ICB combination therapy, anti-mouse PD-1 (Rat IgG2a; Bioxcell) or anti-mouse PD-L1 (mIgG3e; Invivogen, San Diego, California, United States) mAbs or vehicle were i.p. injected (200 µl; 50 µg/mouse; 2.5 mg/kg) starting on Day 8 or 10 postinoculation (day four of the 1^st^ cycle) and on the 1^st^ and 4^th^ days of the following treatment cycles. Minimal effective ICB dosing was used to avoid fatal hypersensitivity due to repeated xenogeneic αPD-(L)1 administration in the inflammatory MC38 tumor model.

For the immunophenotyping study, 24 h after the last treatment of cycles 1–3 and 96 h after cycle 4, four mice/group were sacrificed. It is noteworthy that by the initiation of treatment cycle 4, all mice in the control and anti-PD-1 monotherapy groups had reached the study endpoint of tumor size and were not included in the final analysis performed 96 h after the completion of cycle 4 (C4); hence, only C215Fab-SEA and combination treatment group data are displayed for the C4 analysis. Tumor and (inguinal) tumor-draining lymph nodes (TDLNs) were collected in fresh RPMI 1640. TDLNs were placed on ice, and the node capsule was disrupted using forceps to release all leucocytes into a single cell suspension. Cells isolated from TDLNs were further processed and analyzed by flow cytometry (FC) as described below. Tumors were weighed and cut into three samples. Fresh samples were taken for FC analysis, and the remaining tissue was snap frozen for IHC analyses or stored in RNALater (Invitrogen, Waltham, Massachusetts, United States) for TCR and NanoString RNA analyses.

All other mice were monitored for tumor growth and survival and were sacrificed when moribund or when the tumor volume was > 2500 mm^3^ according to the ethically approved protocol. On Day 75 or 100 after the initial tumor inoculation, tumor-free mice were rechallenged with 500,000 MC38-hEpCAM cells in the same flank as the initial inoculation and 500,000 MC38 parental cells in the contralateral flank.

For adoptive cell transfer, T cells were isolated from the spleens of tumor-free (TF) mice that were resistant to a second challenge (n = 3) or naïve littermates (n = 6) on Day 150 after the initial tumor inoculation using the mouse Pan T-cell Isolation Kit II (Milteneyi Biotech, Bergisch Gladbach, Germany) according to the manufacturer’s instructions. Isolated T cells from TF mice were i.v. injected (5 × 10^6^ T cells/mouse) into 13, 7–8-week-old C57BL/6 J female mice, and isolated T cells from naïve mice were i.v. injected (5 × 10^6^ T cells/mouse) into six 7–8-week-old C57BL/6 J female mice 48 h before MC38-hEpCAM tumor cell inoculation.

Tumor growth was monitored by measuring perpendicular tumor diameters with calipers. Tumor volume was calculated using the following formula: V = (D × d^2^)/2, in which V is the volume (mm^3^), D is the larger diameter (mm), and d is the smaller diameter (mm). Mouse weight was recorded and monitored biweekly over the course of the in vivo studies.

### Flow cytometry (FC)

For FC, tumor tissues (50–500 mg) were sliced into small fragments and dissociated into single cells using the mouse Tumor Dissociation Kit (Milteneyi Biotech), gentleMACS C tubes, and the gentleMACS Dissociator (Soft/medium 37C_m_TDK_1 program). Inguinal TDLNs (1 TDLN/specimen) were placed on ice and minced using forceps to release immune cells, washed, and passed through a 40 µm cell strainer before further processing for FC.

Single-cell suspensions from tumors and TDLNs were stained for viability (Zombie NIR; BioLegend, San Diego, California, United States), washed, and incubated with TruStain fcX (Clone 93, BioLegend) for 10 min at room temperature (RT). Without washing, the cells were incubated with antibodies for 30 min at RT. Antibodies for FC were from Milteneyi Biotech or BioLegend unless indicated otherwise. Monoclonal anti-mouse antibodies from BioLegend were as follows: Rat CD90.2- PerCP-Cy5.5 (clone 53–2.1), mouse NK1.1-PE/Cy7 (clone PK136), rat CD4-FITC (clone GK1.5), rat CD45-Pacific blue (clone 30-F11), rat CD11b-APC (clone M1/70), Armenian hamster CD11c-PE/Cy7 (clone N418), rat CD206-PerCP-Cy5.5 (clone C068C2), rat CD127-APC (clone A7R34), Armenian hamster CD103-Briliant violet 510 (clone 2E7), rat CCR7- PerCP-Cy5.5 (clone 4B12), Armenian hamster IgG-APC (clone HTK888), Armenian hamster IgG-PE/Cy7 (clone HTK888), rat IgG2a-PerCP-Cy5.5 (clone RTK2758), rat IgG2a-APC (clone RTK2758), and mouse IgG1-APC (clone MOPC-21). Monoclonal antibodies from Milteneyi Biotech were as follows: CD137-APC (clone 17B5-1H1), TCR V beta 3-PE (clone REA646), CD8-Viogreen (clone 53–6.7), MHC II-FITC (clone REA813), Gr-1-Viogreen (clone REA810), CD25-APC (clone 7D4), Foxp3-PE (clone REA788), REA-FITC (clone REA293), Rat IgM-APC (clone ES26-13D3.4), REA-PE (clone REA293), and F4/80-PE (clone Cl:A3-1; Serotec Bio-Rad, Oxford, United Kingdom). Samples were measured on a MACSquant V cytometer (Milteneyi Biotech), and data were analyzed using FlowJo version X.0.7 (Tree Star, Inc. Ashland, OR, US). Flow cytometry gating strategies for T cells and antigen-presenting cells (APCs) are shown in Extended Data Fig. 1.

**Fig. 1:**
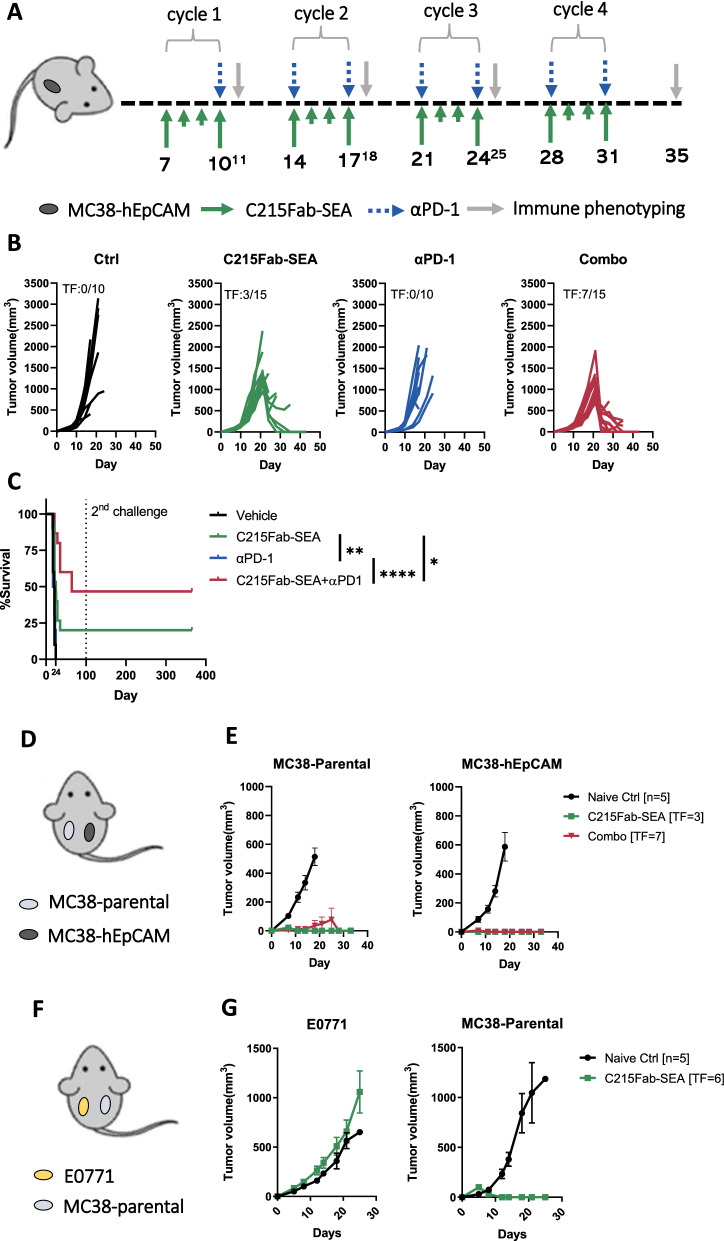
C215Fab-SEA significantly inhibited tumor growth, increased survival and induced a broad protective polyclonal immune response against tumor rechallenge. **A** Schematic illustration of the dosing regimens for the in vivo study. Mice were subcutaneously (s.c.) injected with 5X10^5^ MC38-hEpCAM tumor cells and were randomized on Day 7 (≈ 60 mm^3^ mean tumor volume) into treatments of C215Fab-SEA (15 μγ/mouse; i.p.), anti-PD1 mAb (50 μγ /mouse; i.p.) or combined therapy. After each treatment cycle, tumors were taken for immune phenotyping analysis. **B** Individual tumor growth kinetics of mice from the control and treated groups. TF = Tumor free. **C** Kaplan‒Meier overall survival curves of each treatment group. Survival data were evaluated for statistical significance with the log-rank Mantel‒Cox test. *p < 0.05; **p < 0.01; ****p < 0.0001. **D** On Day 100 after tumor inoculation, TF mice were rechallenged with 5 × 10^5^ MC38-hEpCAM injected (s.c.) in the right flank and challenged with 5 × 10^5^ MC38 parental cells (hEpCAM negative) in the left flank. The hEpCAM negative challenge tests whether the protective memory is broad and polyclonal, as hEpCAM is the target of C215Fab-SEA. For comparison, naïve mice were also challenged with both cell lines. **E** Mean (± SEM) tumor volume of MC38-parental (left) or MC38-hEpCAM (right) in naïve mice (black), TF mice of C215Fab-SEA monotherapy (green) and TF mice of combination therapy (red). **F** On Day 100 after tumor inoculation, TF mice were rechallenged with 5X10^5^ MC38 parental cells injected (s.c.) in the right flank and challenged with 2.5 × 10^6^ E0771 cells in the left flank. The challenge tests whether the protective memory is specific to MC38-associated antigens. For comparison, naïve mice were also challenged with both cell lines. **G** Mean (± SEM) tumor volume of E0771 breast tumor (left) or MC38-parental colon (right) in naïve mice (black) and TF mice of C215Fab-SEA monotherapy (green). In all figures, timepoints are referred to as C1-C4 (cycles 1-4, respectively). Data are representative of at least 3 independent experiments

### Tumor RNA isolation

Frozen tumors were stored in RNALater at – 20 °C until further processing. Tumor tissues were thawed, RNALater was removed, tissue was homogenized in TRI Reagent® (MERCK, Kenilworth, New Jersey, United States), and RNA was extracted according to MERCK's instructions. RNA concentrations were measured using a NanoDrop 1000 Spectrophotometer (Thermo Scientific Waltham, Massachusetts, United States), and RNA integrity was analyzed using the RNA ScreenTape and 2200 TapeStation system (Agilent Technologies, Santa Clara, CA, United States).

### NanoString and computational analysis

The NanoString panel (Mouse PanCancer IO360, v1.0) was used to assess immune gene signature scores in MC38-hEpCAM tumors following each treatment cycle. T-cell signature gene set descriptions can be found in Additional file 5: Table S1. Gene expression was quantified from the total RNA of each tumor sample with the NanoString nCounter platform using 200 ng of total RNA for the nCounter Mouse IO360 Panel, comprising 770 immunology-related mouse genes (NanoString Technologies). The code set was hybridized with the RNA overnight at 65 °C. RNA transcripts were immobilized and counted using the NanoString nCounter Digital Analyzer. The lower limit of detection is 20 counts, and thus, transcripts for which over 95% of samples had counts under 20 were excluded from subsequent analysis. Gene expression normalization was performed relative to housekeeping genes using the GeoMean algorithm. Of the 20 housekeeping genes included in the panel, 13–15 with the lowest standard deviations after normalization were used (SD < 0.45), using the geNorm algorithm for each comparison. Mean square error analysis was used to identify potential samples of low data quality, and no samples were removed. All normalized data were then transformed on a log2 scale for further analysis. Advanced Analysis Immune Cell Type Profiling was performed with nSolver4.0 and Advanced Analysis package 2.0 (NanoString; Seattle, Washington, United States), following the default analysis pipeline software to predict changes in cell type frequencies based on the measured expression of gene sets validated to be cell–type-specific and compared to data independently derived by FC [19].

Selected housekeeper genes used for the normalization of cycle 3 analyses were as follows: *Tmub2, Psmc4, Nrde2, G6pdx, Ubb, Tbp, Mrpl19, Tbc1d10b, Dnajc14, Ercc3, Gusb, Tlk2, Polr2a, Sf3a1, Pum1, Sdha, Abcf1, Tfrc, Oaz1,* and* Stk11ip.*

### TCRβ CDR3 region sequencing and repertoire analysis

Purified RNA samples were quantified using a Qubit RNA HS Assay Kit (Thermo Fisher Scientific). For each sample, 200 ng DNA was amplified using the Ion AmpliSeq™ Mouse TCR Beta SR RNA Assay (Thermo Fisher Scientific) according to the manufacturer’s instructions. Libraries targeting CDR3 regions were purified with Agencourt AMPure XP beads (Beckman Coulter, Brea, California, United States), washed with 70% ethanol, and eluted in 50 μL Low TE buffer. The resulting library samples were quantified using the Ion Library Quantitation Kit (Thermo Fisher Scientific) and diluted to 25 pM with Low TE buffer. Equal volumes of 15 samples, including the control, were pooled together for sequencing on a 540 Chip using an Ion Torrent S5XL sequencer, followed by analysis via Ion Reporter version 5.14.1 (Thermo Fisher Scientific). Ion AmpliSeq Mouse TCRB-SR—w1.2—RNA—Single Sample was utilized to estimate TCRβ metrics of clone frequencies, diversity, evenness, clonality, and convergence. Clone frequency was calculated as the frequency of a unique VDJ rearrangement as a proportion of total reads passing quality filtering. TCRβ repertoire diversity was calculated as Shannon’s entropy, where diversity = -$\sum_{i=1}^{R}{p}_{i} log2 ({p}_{i})$. Here, ${p}_{i}$ indicates the frequency of the i^th^ clone, and R indicates the total number of clones. Evenness is a measurement of the similarity of clone sizes. Evenness = $\frac{\sum_{i=1}^{R}{p}_{i} log2 ({p}_{i}) }{log2 (R)}$, where ${p}_{i}$ indicates the frequency of the i^th^ clone and R indicates the total number of clones. Evenness ranges from 0 to 1. Samples with clones of equal frequency will have an evenness of 1, whereas samples with clones of unequal sizes will have an evenness of less than 1. Clonality is calculated as 1-evenness. The convergence of TCRβ was calculated as the frequency of clonotypes that are identical in amino acid sequence but different in nucleotide sequence. RNA processing, TCR amplification, TCRβ sequencing and TCR repertoire analysis were all performed by Omniseq (Buffalo, NY, USA).

### Immunohistochemistry

Cryosectioned (8 μm) tissues (n = 2–3/group/cycle) were stained for CD3 or CD45. After fixation, the sections were preblocked in normal mouse serum for 20 min, followed by incubation with primary antibodies for 1 h and secondary antibodies for 30 min. To visualize staining, slides were incubated with ready-to-use polymer BrightVision anti-rabbit/HRP for 30 min, and color was developed with diaminobenzidine (DAB) for 5 min. Slides were counterstained with hematoxylin, dehydrated, and mounted with coverslips. Analyses and microphotography were performed using a Leica DMRX microscope equipped for light microscopy. For double staining, cryosections were fixed and preblocked in normal mouse serum for 20 min. Cryosections were incubated with primary antibodies for 2 h (for each double labeling, cryosections were incubated with both antibodies simultaneously), after which they were incubated with secondary antibodies for 30 min. Slides were mounted with fluorescence mounting media and coverslips. Primary antibodies and dilutions were as follows: CD3 (KT3, 1:200; Serotec Bio-Rad), CD45 (13/2.3, 1:150; BioDesign, Carmel Hamlet, New York, United States), Granzyme B-FITC (NGZB, 1:500; Invitrogen), CD8-Viobright FITC (REA601, 1:15; Milteneyi Biotech), FoxP3-Vio667 (REA788, 1:15; Milteneyi Biotech), CD4-FITC (RM4-5, 1:100; eBioscience, San Diego, California, United States), Gr-1-FITC (REA810, 1:15; Milteneyi Biotech), and CD11b-FITC (M1/70, 1:500; Thermo Scientific). The secondary antibodies were rabbit anti-rat IgG (Jackson ImmunoResearch, 1:600; Baltimore Pike, United States), goat anti-human Alexa Fluor 555 (Invitrogen, 1:500), and goat anti-human Alexa Fluor 488 (Invitrogen, 1:500). All analyses and microphotography were performed using a Leica DMRX microscope equipped for fluorescence microscopy. All IHC staining was performed by Micromorph (Lund, Sweden).

### Statistical analysis

Statistical analyses were performed using GraphPad Prism (GraphPad 9.3.1 Software Inc.). For survival analyses, Mann‒Whitney unpaired tests were performed to assess differences between the median survival times of the relevant groups. Tumor growth was assessed using two-way ANOVA with a Bonferroni post hoc test. Error bars in figures indicate the mean plus or minus the standard error of the mean (SEM). Overall, a p value < 0.05 was considered statistically significant: *p ≤ 0.05, **p ≤ 0.01, ***p ≤ 0.001, and ****p ≤ 0.0001. All statistical analyses on NanoString data were performed on log2 transformed normalized counts. Differential expression analyses were carried out using nSolver4.0 and the Advanced Analysis package 2.0 to determine differentially abundant transcripts with a preset threshold of statistical significance. To control for multiple testing, an adjusted p value (i.e., false discovery rate (FDR) q-value) threshold of 0.01 or 0.05 was used for statistical significance.

## Results

### C215Fab-SEA induces antitumor activity and the generation of sustainable, long-lasting protective memory against tumors in vivo

To study the effect of TTS on the long-term activation of T cells against tumors, we tested the in vivo efficacy of C215Fab-SEA as monotherapy and in combination with ICB therapy in the MC38 tumor model. The experimental design is illustrated in Fig. 1A and Additional file 2: Fig. S2A. In this model setting, the MC38-hEpCAM tumors grew progressively in control mice, and anti-PD-1 monotherapy had a limited effect (Fig. 1B, Additional file 2: Fig. S2B). Mice treated with C215Fab-SEA alone experienced inhibited tumor growth and prolonged survival, and complete tumor clearance was observed in 10–20% of treated mice (Fig. 1B, C, Additional file 2: Fig. 2B, C). The effects of C215Fab-SEA monotherapy were further enhanced when combined with anti-PD-1, which resulted in significantly improved tumor growth inhibition and higher rates of tumor-free mice (Fig. 1B, C, Additional file 2: Fig. 2B, C). Similar results were observed when C215Fab-SEA was combined with anti-PD-L1 (Additional file 3: Fig. S3B).

**Fig. 2:**
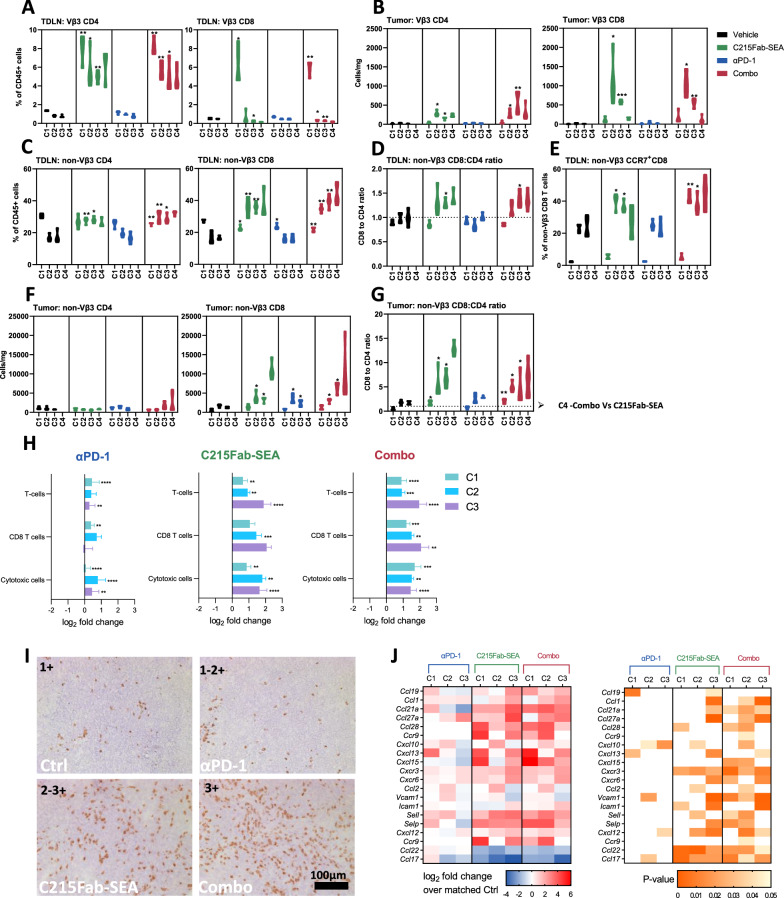
C215Fab-SEA treatment induced a rapid T-cell influx into TDLNs and enhanced T-cell infiltration into the TME. **A** Violin plots showing the percentage of Vb3 CD4 (left) or CD8 (right) T cells of total CD45^+^ cells in the TDLNs as determined by FC. **B** Violin plots showing the cell density (cells/mg tumor tissue) of Vβ3 CD4^+^ (left) or CD8^+^ (right) T cells in tumors as determined by FC. **C** Violin plots showing the percentage of non-Vβ3 CD4 (left) or CD8 (right) T cells among total CD45 + cells in the TDLNs as determined by FC. **D** Non-Vβ3 CD8^+^/CD4^+^ T-cell ratios in TDLNs. **E** Violin plots showing the percentage of CCR7 + cells among non-Vβ3 CD8 T cells in the TDLNs. **F** Violin plots showing the cell density of non-Vβ3 CD4 (left) or CD8 (right) T cells in the TME. **G** Violin plots showing the CD8/CD4 T-cell ratios of non-Vβ3 cells found in the TME. **A**–**G** FC analysis; n = 3–4/group/cycle; timepoints are referred to as C1-C4 (cycles 1–4, respectively). Statistical significance was determined by two-way ANOVA with Tukey’s multiple comparisons test. *p < 0.05, **p < 0.01, ***p < 0.001, ****p < 0.0001, versus the timepoint-matched control; for C4 Combo versus C215Fab-SEA **H** Graphs displaying the mean (± SEM) log2-fold-change of immune populations according to their gene signatures (Additional file 5: Table S1) at each treatment cycle compared to a matched control group in tumors. n = 3–4/group/cycle. Statistical significance was determined by nSolver software **I.** Representative immunohistochemical (IHC) CD3 staining analysis of frozen sections (n = 2–3/group/cycle) of MC38-hEpCAM tumors from the control and treatment groups 24 h after the completion of cycle 3. Scale bar, 100 µm; IHC score (upper right): 1 +  = few positive cells, 1–2 +  = few to moderate numbers, 2 +  = moderate numbers, 2–3 + moderate to high numbers, 3 + high numbers. **J** Heatmap displaying the relative (mean log2-fold-change) expression of selected chemokine and adhesion genes associated with T-cell infiltration and chemotaxis compared to a matched control group in the TME (left). n = 3–4/group/cycle. The color gradient indicates the fold-change over the matched control. The right heatmap shows the p values of the significant differences in the left heatmap. White represents a nonsignificant expression change compared to the matched control. Differential expression analysis was performed with Welch’s t test

Mice that were tumor-free (TF) after either C215Fab-SEA monotherapy or C215Fab-SEA and ICB combination therapy were resistant to rechallenge with MC38-hEpCAM. While 100% of the naïve mice developed flank tumors on both sides, all TF mice completely rejected the second tumor challenge. All the naïve mice died by Day 24 of the study, whereas all the TF mice survived for at least 365 days after rechallenge with no recurrence of the tumors. Remarkably, without any further treatment, these mice were also resistant to challenge with MC38 parental tumors, demonstrating a long-lasting protective memory that is not dependent on the presence of the tumor-specific antigen originally targeted by the TTS (Fig. 1D, E, Additional file 2: Fig. S2D, Additional file 3: Fig. S3C, D). Moreover, while tumor-free mice previously treated with C215Fab-SEA monotherapy were resistant to MC38 parental colorectal tumors, they failed to reject the E0771 breast tumor cells injected in the contralateral flank (Fig. 1F). These results indicate that the acquired protective memory was driven by Ag-specific responses directed against MC38-associated antigens. We further tested the extent and sustainability of the protective memory responses of pan-T cells isolated from treated tumor-free mice and transferred them into MC38-bearing naïve mice (Additional file 3: Fig. S3E). In line with our previous results, the transfer of these T cells into naïve mice induced protective memory and prevented tumor growth in 11 out of 13 mice, while mice that received T cells derived from untreated mice failed to show tumor growth control (Additional file 3: Fig. S3F).

These results indicate that C215Fab-SEA treatment synergizes with ICB to achieve a strong antitumor response and highlight the potency of C215Fab-SEA as monotherapy and in combination with an ICB agent to drive T-cell-dependent protective immunity long after tumor clearance.

### C215Fab-SEA induces increased T-cell migration and infiltration

The effects of the TTS alone and in combination with anti-PD-1 on T-cell migration and infiltration were first tested in the poorly immunogenic B16F10 lung metastasis tumor model. Similar to the MC38 study, in this model, the administration of C215Fab-SEA together with anti-PD-1 provoked a synergistic antitumor response, resulting in markedly improved long-term survival (Additional file 4: Fig. S4A, B). IHC analysis of lung tumors following C215Fab-SEA treatment alone and in combination with anti-PD-1 treatment was shown to promote GrzB^+^CD8^+^ T-cell infiltration even into the poorly immunogenic B16F10 melanoma tumors, turning “cold” tumors into immunogenically “hot” tumors (Additional file 4: Fig. S4C, D).

To further investigate the effect of TTS on T-cell infiltration and activation and to better understand the mechanism behind the generation and persistence of memory T cells, we performed an immune phenotyping study in the MC38-hEpCAM model. We conducted comprehensive ex vivo analyses over the course of the study, as described in Fig. 1A and the methods. As expected, FC analysis of cells extracted from TDLNs and tumors revealed significantly higher frequencies of TCR Vβ3 CD4^+^ and CD8^+^ T cells following C215Fab-SEA administration than in the control and anti-PD-1 groups (Fig. 2A, B). However, although Vβ3 CD4^+^ T-cell frequencies were high and stable over the course of the four treatment cycles, the numbers of Vβ3 CD8^+^ cells declined markedly in the TDLNs and increased significantly in the tumors by the completion of treatment C2 (Fig. 2A, B). Interestingly, by the completion of treatment C4, Vβ3 CD8^+^ cell numbers had dropped to baseline levels in the tumors (Fig. 2B). As the direct cytotoxic killing of superantigen-coated tumor cells is predominantly executed by Vβ3 CD8^+^ T cells [10], these data suggest that TCR Vβ3 CD8^+^ effector T cells egressed from the TDLNs into the tumor. We suspect that serial engagements of TCR Vβ3 CD8^+^ T cells with superantigen-coated tumor cells ultimately led to decreased numbers of these exhausted CD8^+^ T cells in the TME by the completion of treatment C4. Importantly, Vβ3 T-cell expansion in the TDLNs was also accompanied by elevated frequencies of non-Vβ3 CD8^+^ T-cell clones with increased CD8 to CD4 ratios in the TDLNs of C215Fab-SEA treated groups, indicating the induction of a bystander effect on multiple CD8^+^ T-cell clones residing in the TDLNs (Fig. 2C, D). In line with the increase in frequency, the general CD8^+^ T-cell population in the TDLNs of C215Fab-SEA-treated mice exhibited upregulated CCR7 expression over the treatment cycles (Fig. 2E). Given that CCR7 is a migratory marker for T-cell influx [20, 21], these data indicate enhanced influx of CD8^+^ T cells into the TDLNs through the afferent lymphatic vessels.

In the tumors, C215Fab-SEA treatment also led to a significant increase in non-Vβ3 T-cell frequencies (Fig. 2F), with CD8 to CD4 ratios preferable to those of controls (Fig. 2G). Notably, in contrast to TCR Vβ3 CD8^+^ T-cell frequencies, the frequencies of non-Vβ3 CD8^+^ T cells in the tumor were increased by the completion of C4 (Fig. 2F). Moreover, T-cell gene signatures were also significantly changed over the course of C215Fab-SEA treatment cycles, while during treatment with anti-PD-1 alone, the changes in immune infiltrate frequencies were seen to a lesser extent and mainly at C2 (Fig. 2H). This temporary effect of anti-PD-1 is in line with its limited in vivo effect in this model setting. Interestingly, the combination treatment resulted in higher T-cell scores, indicative of enhanced inflammation and increased cytotoxic cell abundance in the TME (Fig. 2H). The increase in the infiltration of total CD3^+^ T cells in the tumors of the C215Fab-SEA treatment groups was also confirmed by IHC staining (Fig. 2I).

Our data also showed that C215Fab-SEA induced the enrichment of multiple genes that regulate the recruitment of effector T cells into the TME (Fig. 2J). Significant and strong upregulation of major chemokines and chemokine receptors known to attract T cells into tumors (*CC21*, *Cxcr3, Cxcr6*, *Ccl27a, CXCL12* and *Ccl28)* [22–24] and of E-selectin (*Sele*) and P-selectin (*Selp*), which guide the trafficking of activated T cells [25, 26], was detected over the course of treatment in the tumors of the C215Fab-SEA-treated groups, while anti-PD-1 alone had a lesser impact on the mRNA levels of these transcripts (Fig. 2J). This increase in the expression of chemokines and selectins provides further support for the recruitment of activated T cells into the TME following C215Fab-SEA treatments. Interestingly, the onset of the upregulation of most of these genes was detected in the combination group in early stages by the completion of treatment C1 (Fig. 2J), indicating that the advantage of C215Fab-SEA and anti-PD-1 combination therapy is to induce a “hot” immune status in the TME more rapidly than C215Fab-SEA monotherapy.

### C215Fab-SEA treatment remodels the immunosuppressive TME into an immunostimulatory TME

**Fig. 3:**
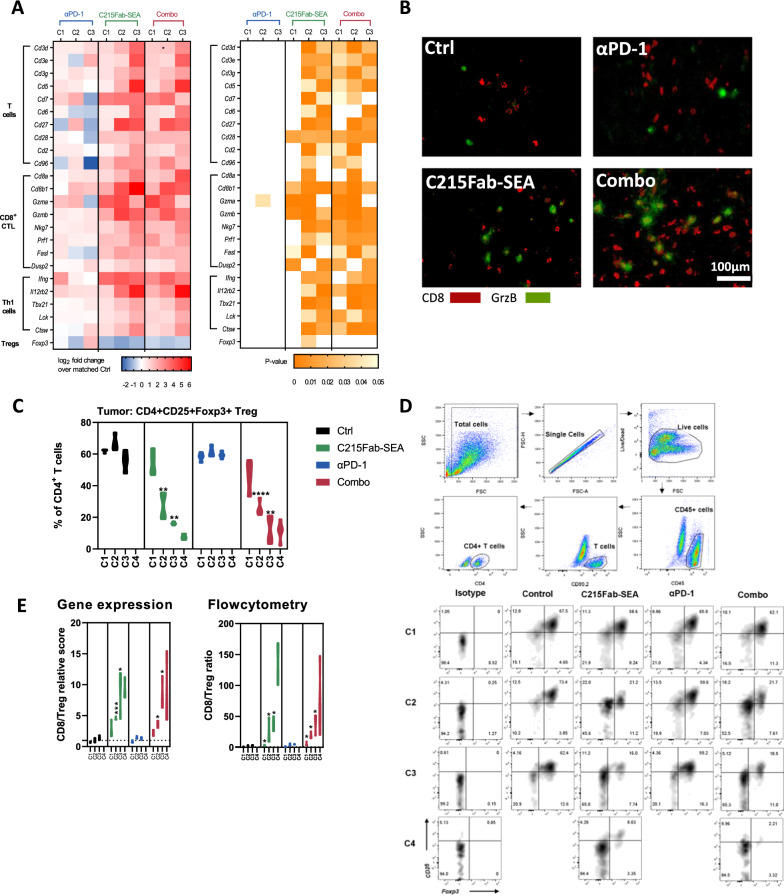
C215Fab-SEA promoted CD8 T-cell cytotoxic function and reduced the Treg cell number in the TME. **A** Heatmap displaying the mean log2-fold-change values of the expression of selected genes associated with T cells in tumors following different treatments (left). n = 3–4/group/cycle. The color gradient indicates the fold increase relative to the designated control. The right heatmap shows the p values of the significant differences in the left heatmap. White represents a nonsignificant expression change compared to the matched control. Differential expression analysis was performed with Welch’s t test. **B** Representative immunohistochemical (IHC) double staining analysis (n = 2–3/group/cycle) of CD8 (red) and granzyme B (green). Frozen sections of MC38-hEpCAM tumors from the control and treatment groups were taken 24 h after the completion of cycle 3 for IHC analysis. Scale bar, 100 µm. **C** Violin plots show the percentage of CD25^+^Foxp3^+^ cells among the total CD4^+^ T cells in the tumors as determined by FC. Statistical significance was determined by two-way ANOVA with Tukey’s multiple comparisons test. n = 3–4/group/cycle. *p < 0.05, **p < 0.01, ***p < 0.001, ****p < 0.0001, versus the timepoint-matched control; for C4 Combo versus C215Fab-SEA. **D** Pseudocolor plots showing Treg gating strategy and representative FC scatter plots (n = 3–4/group/cycle) demonstrating the reduction in Tregs in the TME following C215Fab-SEA. **E** Graph displaying CD8/Treg ratios in the TME according to NanoString gene expression analysis (left) and FC (right) of MC38-hEpCAM tumors after the completion of each treatment cycle. Statistical significance was determined by one-way ANOVA with Tukey’s multiple comparisons test. n = 3–4/group/cycle. *p < 0.05, **p < 0.01, ***p < 0.001, ****p < 0.0001, versus the timepoint-matched control, for C4 Combo versus C215Fab-SEA. In all figures, timepoints are referred to as C1-C4 (cycles 1-4, respectively).

We further explored the effect of the different treatments on T-cell and macrophage populations in the TME. Our gene expression data show that while anti-PD-1 alone had a limited effect on T-cell populations and activation, C215Fab-SEA as monotherapy and in combination with anti-PD-1 induced significant upregulation of multiple genes associated with T cells, such as *Cd3d, Cd3e* and *Cd3g,* and costimulatory genes *Cd28* and *Cd27*. In comparison, anti-PD-1 alone had no significant effect on T-cell populations and activation. In addition, genes involved in Th1 responses and CD8 cytotoxic T-cell (CTL) activity, such as *Ifng, Il12rb2, tbx21, lck, Ctsw, Gzma, Gzmb, Nkg7 and Prf1,* were significantly upregulated in tumors of these groups, indicative of boosted activity of the T cells in the TME (Fig. 3A) [27–29]. Notably, whereas C215Fab-SEA alone induced upregulation of T-cell-related genes, mainly following C2, the combination therapy had a significant effect after C1 (Fig. 3A). Consistently, increased GrzB^+^CD8^+^ T-cell (CTL) infiltration was also shown in IHC double staining of tumor sections (Fig. 3B). Interestingly, our gene expression data also revealed reduced levels of Foxp3 (a gene typically expressed by Tregs) in the tumor following C215Fab-SEA treatments (Fig. 3A). We further investigated this finding by measuring the frequencies of Tregs in the tumors over the course of treatment by FC (Fig. 3C). The results revealed that C215Fab-SEA treatments induced a significant decrease in Treg frequency, whereas high frequencies of Tregs were found in the control and anti-PD-1 groups (Fig. 3C, D). A major mechanism supporting Treg tissue abundance is chemoattraction [30, 31], and among the chemokines that attract and activate Tregs, CCL17 and CCL22 may be particularly important [32–34]. Our results showed a significant decrease in CCL17 and CCL22 transcripts in tumors treated with both C215Fab-SEA-treated regimens, which may explain our findings (Fig. 2J). Finally, the analysis of the immune cell subsets using both FC and gene expression data showed that C215Fab-SEA treatments induced higher CD8^+^ T cell/Treg ratios, while lower ratios were found in tumors treated with anti-PD-1 and the controls (Fig. 3E).

**Fig. 4:**
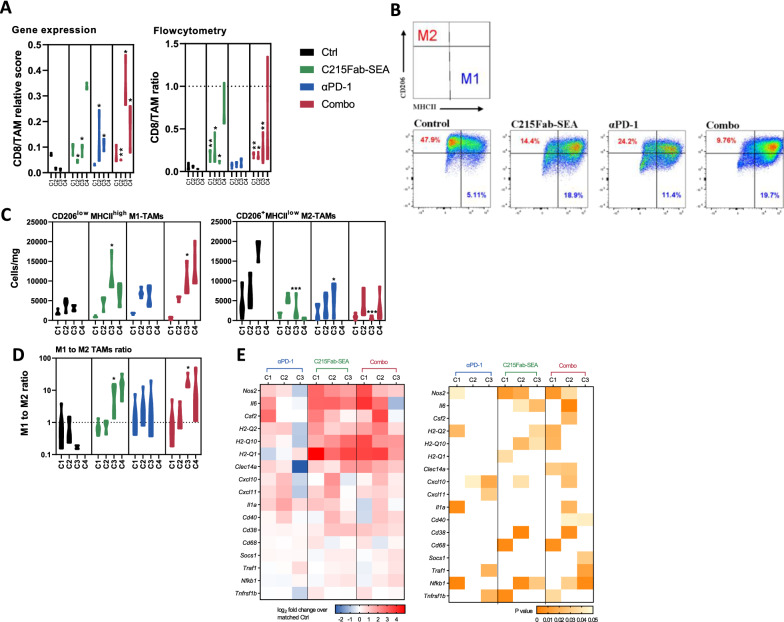
TTS treatments increased the number of M1 macrophages and CD8 T-cell abundance and activity. **A** Graph displaying CD8/TAM ratios in the TME of MC38-hEpCAM tumors after the completion of each treatment cycle, according to FC analysis (left) and NanoString gene expression analysis (right). The TAM (CD11B^+^CD11C^−^F4/80^+^**)** FC gating strategy can be found in Additional file 1: Fig. S1B. **B** Representative pseudocolor plots showing MHC II and CD206 expression in CD11b^+^ F4/80^+^ TAMs on treatment cycle 3 as determined by FC for the detection of TAM M1 and M2 subsets as described in the upper left legend. **C** Violin plots show the tumor density (cells/mg tumor) of CD206^low^MHCII^high^M1-TAMs (left) and CD206^high^MHCII^low^M2-TAMs (right) over the treatment cycles as determined by FC. **D** Violin plots show the tumor M1 to M2 TAM ratios over the study period. **A**–**D** n = 3–4/group/cycle; timepoints are referred to as C1-C4 (cycles 1–4, respectively). Statistical significance was determined by two-way ANOVA with Tukey’s multiple comparisons test. *p < 0.05, **p < 0.01, ***p < 0.001, ****p < 0.0001, versus the timepoint-matched control, for C4 Combo versus C215Fab-SEA. **E** Heatmap displaying the mean log2-fold expression values of changed genes (left) associated with M1 macrophage activity in tumors upon different treatments over the treatment cycles. n = 3–4/group/cycle. The color gradient indicates the fold increase relative to the matched control. White represents a nonsignificant expression change compared to the matched control. Differential expression analysis was performed with Welch’s t test

In addition to Tregs, tumor-associated macrophages (TAMs) are also major players in inducing the immunosuppressive microenvironment associated with many tumors. TAMs represent one of the main tumor-infiltrating immune cell types and are generally categorized into M1 macrophages, which exert antitumor functions, and M2 macrophages, which contribute to tumor progression [35–37]. Both FC and gene expression analyses showed significantly improved CD8^+^ T-cell/TAM ratios following C215Fab-SEA treatments but a lesser extent of improvement with anti-PD-1 monotherapy (Fig. 4A). Furthermore, based on the surface expression of CD206 and MHC II upregulation, FC analysis revealed that by C3, favorable M1/M2 ratios were detected in all three treatment groups compared to the control group (Fig. 4B–D). C215Fab-SEA treatments increased CD206^low^MHCII^high^M1-TAM frequencies while decreasing CD206^high^MHCII^low^M2-TAM frequencies. This is in line with gene expression analysis showing an increase in M1-related genes in the treatment groups (Fig. 4E) [37, 38].

Collectively, our results show that treatment with C215Fab-SEA induces T-cell infiltration into tumors, with more CD8 cytotoxic T cells and M1 macrophages and fewer Tregs and M2 suppressive TAMs. The combination of C215Fab-SEA with anti-PD-1 induced early increases in T-cell abundance and function in the TME, allowing more profound immune stimulation by C215Fab-SEA and at earlier treatment stages. The increase in the M1/M2 ratio following both anti-PD-1 monotherapy and C215Fab-SEA treatments provides further proinflammatory support for the T-cell responses in the TME.

### C215-Fab-SEA induces epitope spreading and promotes T-cell memory differentiation in tumors

**Fig. 5:**
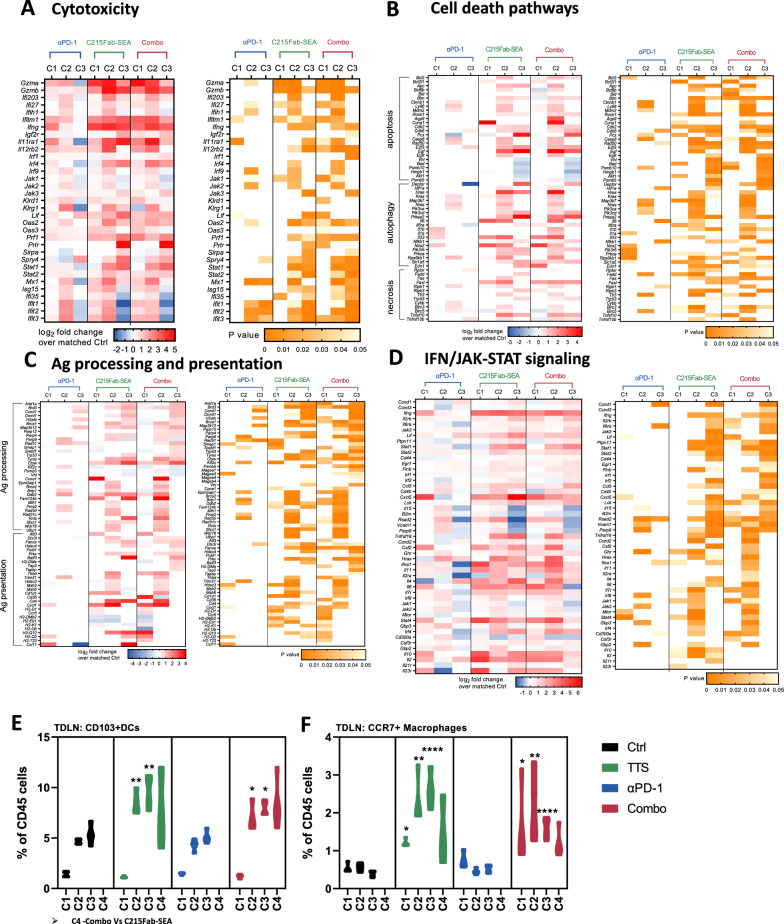
T-cell polyclonal expansion is derived by effective Ag uptake and epitope spreading via CD103 + cross-presenting DCs and macrophages **A**–**D** Heatmaps displaying the mean log_2_-fold expression values of significantly changed genes (left; p < 0.05) in tumors upon different treatments over the treatment cycles (C1–C3). The color gradient indicates the fold increase relative to the matched control. The right heatmap shows the p values of the significant differences in the left heatmap. White represents a nonsignificant expression change compared to the matched control. Differential expression analysis was performed with Welch’s t test. n = 3–4/group/cycle. **A** Genes associated with cytotoxicity in tumors. **B** Genes associated with cell death processes—apoptosis (top), autophagy (middle) and necrosis (bottom). **C** Genes associated with antigen processing and presentation in tumors. **D** Genes associated with interferon and JAK-STAT signaling. **E** Violin plots show the % of CD103^+^cDC1 cells found in TDLNs out of the total CD45 + cells over the tested timepoints as determined by FC. The cDC1 (CD103^+^CD11C^+^CD11b^−^) flow cytometry gating strategy can be found in Additional file 1: Fig. S1B. **F** Violin plots show the % of macrophages detected in TDLNs out of the total CD45^+^ cells over the tested timepoints as determined by FC. **E**, **F** n = 3–4/group/cycle; timepoints are referred to as C1-C4 (cycles 1–4, respectively). Statistical significance was determined by two-way ANOVA with Tukey’s multiple comparisons test. *p < 0.05, **p < 0.01, ***p < 0.001, ****p < 0.0001, versus the timepoint-matched control, for C4 Combo versus C215Fab-SEA

Cumulatively, our data suggest that the enhanced T-cell responses in the TME following C215Fab-SEA treatment drive the death of tumor cells. C215Fab-SEA induced significant upregulation of genes associated with cytotoxicity, such as *Gzma, Gzmb,* and *Prf1* (Figs. 3A,  5A), and tumors were enriched with CTLs (Fig. 3B). In addition, our gene expression analysis showed profound induction of cell death processes [39–41] with repeated injections of C215Fab-SEA, while only low-to-moderate upregulation of these gene signatures was obtained with anti-PD-1 monotherapy (Fig. 5B). We hypothesized that tumor cell death leads to epitope spreading [42, 43] and the release of tumor-associated antigens (TAAs) that may be taken up locally by APCs, such as macrophages and CD103 + DCs [44, 45]. Indeed, our analysis revealed significant expression changes in many genes related to both Ag processing and MHC peptide presentation upon C2515Fab-SEA treatment and to a much lower extent upon anti-PD1 monotherapy (Fig. 5C). Furthermore, when examining the expression of genes associated with IFN signaling as well as JAK-STAT genes, we found that C215Fab-SEA treatments induced a cytokine milieu that upregulated MHC class I expression as well as antigen processing and presentation on cells (Fig. 5D) [46, 47]. Moreover, FC analysis of the cells found in the TDLNs of C215Fab-SEA-treated mice showed increased frequencies of the conventional dendritic cell type-1 subset (cDC1) over the course of treatments (Fig. 5E). This is in line with the literature reports that superantigens induce DC maturation in vivo [48, 49] and that migratory CD103^+^cDC1 cells display a robust ability to activate naïve CD8^+^ T cells in LNs and are required to induce a CTL response against tumors [44, 50, 51]. We also detected that C215Fab-SEA treatment induced the influx of migratory CCR7^+^F4/80^+^ macrophages into TDLNs (Fig. 5F), suggesting that Ag-loaded macrophages and DCs are recruited to TDLNs, where they cross-present antigens to antigen-specific T cells. These data demonstrate that repeated C215Fab-SEA injections lead to the priming of a broader antitumor T-cell response by triggering an increased and sustained influx of migratory Ag-presenting cells to TDLNs. This effect was not observed with anti-PD-1 monotherapy (Fig. 5E, F).

**Fig. 6:**
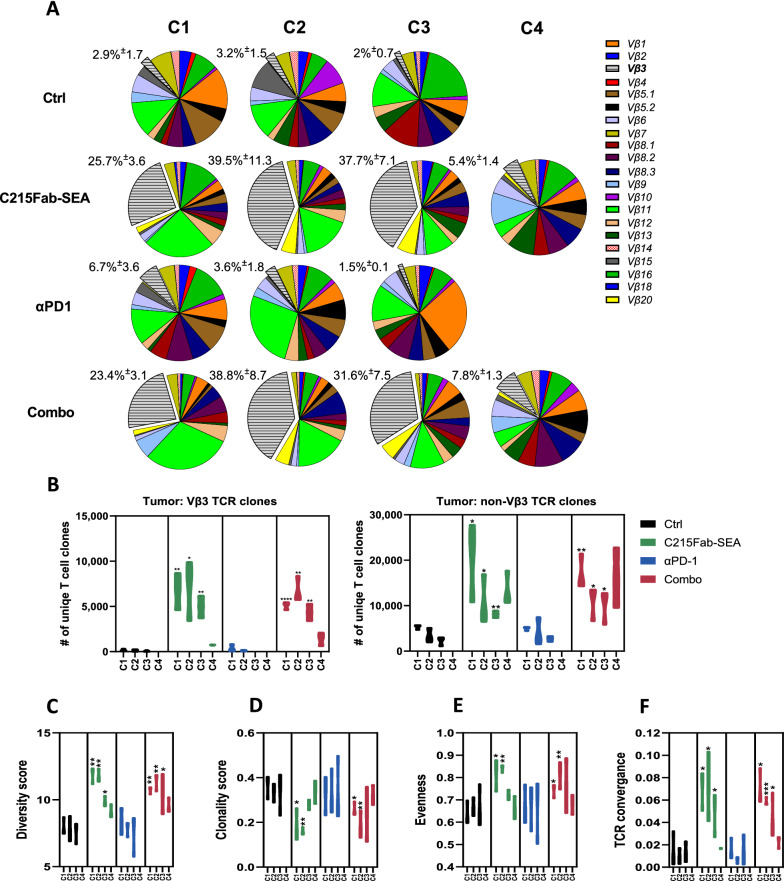
C215Fab-SEA enriched the TCR repertoire diversity in the TME. **A** V beta segment usage pie charts of tumors following treatment at the tested timepoints over the course of the treatment cycles. Pie charts display the mean clonal distribution of each beta chain as detailed in the legend. The frequencies of TCR Vβ3 (gray) are shifted out of the pie chart (n = 3/group/cycle); each pie chart displays > 99% of total TCR β reads. **B** Violin plots display the mean unique TCR Vβ3 (left) and non-TCR Vβ3 (right) T-cell clone counts found in the tumors of each treatment group over the treatment cycles. **C** Violin plot displays the diversity of each sample as calculated by Shannon's entropy (H) index in the tumors over the tested timepoints. Entropy was calculated by summing the frequency of each clone times the log2 of the same frequency over all productive reads in a sample. The higher the H index was, the more diverse the CDR3 clone distribution. **D** Samples were analyzed for the shared occurrence of TCR beta sequences to determine the clonality score. The violin plot shows the clonality score over the treatment cycles in tumors of treated groups defined as the probability of two independently identified sequences originating from the same clone. **E** Violin plot displays the mean (± SEM) evenness score calculated as the relative abundance of unique TCR sequences of each sample using Pielou’s index. Increased evenness indicates the clonal expansion and dominance of TCR clones in the sample. **F** Violin plot displaying TCR convergence in tumors over the tested timepoints. TCR convergence is calculated as the aggregated frequency of clones with unique TCR beta sequences sharing a variable gene and CDR3AA sequence with at least one other identified clone. The timepoints in all figures are indicated as C1-C4 (cycles 1–4, respectively). n = 3/group/timepoint. **B**–**F** Statistical significance was determined by two-way ANOVA with Tukey’s multiple comparisons test. *p < 0.05, **p < 0.01, ***p < 0.001, ****p < 0.0001, versus the timepoint-matched control, for C4 Combo versus C215Fab-SEA

To further explore the “epitope spreading” phenomenon, we conducted TCR sequencing of bulk RNA extracted from tumors. Consistent with our FC results, an increase in TCR Vβ3 usage of up to ~ 40% of total TCR counts was detected in C215Fab-SEA-treated tumors over C1-3, with a decrease at C4 (Fig. 6A). Next, we studied the clonality and diversity of the TCRs based on complementarity determining region 3 (CDR3) sequences. Our results reveal that the number of unique TCR Vβ3 clones found in tumors treated with C215Fab-SEA increased up to 30-fold over the control, indicating a continuous deployment of TCR Vβ3 T-cell clones over C1-3 rather than local expansion of activated resident TCR Vβ3 T cells (Fig. 6B). Importantly, our results also showed an increase in the number of unique TCR clones in non-Vβ3 cells (Fig. 6B). The increase in the number of unique non-Vβ3 clones implies a bystander effect that can occur following epitope spreading. Consistently, the Shannon diversity indices of the C215Fab-SEA-treated tumors were higher than those of the control and anti-PD-1 groups (Fig. 6C). These results suggest that C215Fab-SEA induced the emergence of large numbers of new T-cell clones, resulting in a relatively increased richness of the TCR repertoire under treatment. An increasing proportion of the new clones with selectivity for the tumor is highly probable since MC38 tumor immunity was confirmed (Fig. 1C–E).

**Fig. 7:**
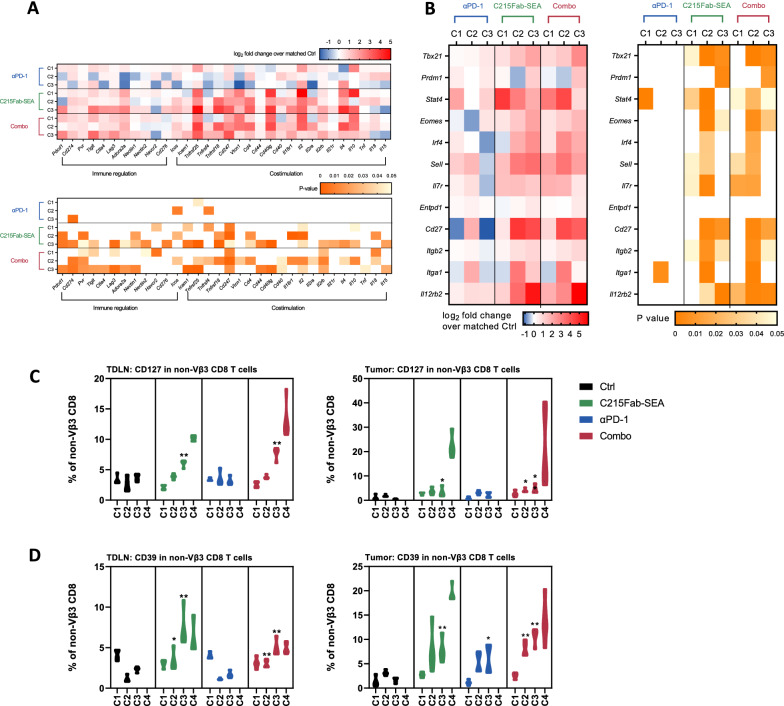
C215Fab-SEA induced increased frequencies of Ag-specific memory T cells. **A**, **B** Heatmaps displaying the mean log_2_-fold expression values of selected genes of T-cell costimulatory and coinhibitory receptors (A-up) and genes associated with T-cell exhaustion and memory development (B-left) in tumors upon different treatments over the treatment cycles (C1–C3). The color gradient indicates the fold increase relative to the matched control. The right (A) or bottom **B** heatmaps show the p values of the significant differences in the up (**A**) or left (**B**) heatmaps, respectively. White represents a nonsignificant expression change compared to the matched control. Differential expression analysis was performed with Welch’s t test. n = 3–4/group/timepoint. **C** Violin plots showing the percentage of CD127-expressing cells among total non-Vβ3 CD8 + T cells in the TDLNs (left graphs) and tumors (right graphs). **D** Violin plots showing the percentage of CD39-expressing cells among the total non-Vβ3 CD8^+^ T cells in the TDLNs (left graphs) and tumors (right graphs). The timepoints in all figures are indicated as C1-C4 (cycles 1–4, respectively). n = 3–4/group/timepoint. Statistical significance was determined by two-way ANOVA with Tukey’s multiple comparisons test. *p < 0.05, **p < 0.01, ***p < 0.001, ****p < 0.0001, versus the timepoint-matched control, for C4 Combo versus C215Fab-SEA

Importantly, although the number of unique non-Vβ3 clones remained high after C4 in the C215Fab-SEA-treated groups (Fig. 6B), the Shannon diversity indices were decreased after C4, presumably due to clonal expansion of non-Vβ3 tumor-reactive T-cell clones (Fig. 6C). This is consistent with the low clonality scores (the probability that two sequence reads are of the same clone; Fig. 6D) recorded over time that increased following C4 of C215Fab-SEA treatment, as well as the decreased clonal evenness scores recorded after C4, which indicate clonal expansion (Fig. 6E). Moreover, we found that different T-cell clones have the same TCR at the protein level, as measured by the TCR convergence score (Fig. 6F). The increased frequency of convergent TCRs within a repertoire, which was revealed in the C215Fab-SEA groups after C1, C2 and C3, is likely to be antigen specific [52, 53]. The increase in TCR convergence scores provides an additional indication of the emergence of MC38-EpCAM tumor immunogenicity and of the T-cell responses to tumor neoantigens induced by C215Fab-SEA but not by anti-PD1. The fact that the convergence index decreased after C4 suggests that at this stage, tumor-specific clones became dominant. Overall, the increased diversity of the T-cell repertoire with gradual elevation in non-Vβ3 TCR clones supports our assumption that C215Fab-SEA treatment induces epitope spreading, which leads to broad T-cell activation against the tumor that is not mediated by the interaction of TTS with the tumor. Notably, TCR analyses revealed that anti-PD-1 monotherapy had no effect on the TCR repertoire. Furthermore, the effect of C215Fab-SEA monotherapy on the TCR repertoire was comparable to the effect of the combination treatment. These results suggest that anti-PD-1 does not drive TCR repertoire diversity in our model. In addition to the increase in the T-cell repertoire, our gene expression data showed that tumors treated with C215Fab-SEA alone or with anti-PD-1 were significantly enriched with both costimulatory transcripts (e.g., *Icam1, Tnfrsf25, Tnfrsf4 (Ox40), Tnfrsf18 (Gitr)*) and coinhibitory transcripts (e.g., *Pdcd1 (Pd1) Cd274 (Pdl1), Tigit, Lag3*), whereas only modest changes were detected following anti-PD-1 upregulation of these costimulatory/inhibitory receptors indicates that C215Fab-SEA treatment drives T-cell stimulation, which is coupled with a commitment to effector and memory cell differentiation [54]. Moreover, transcription factors (TFs) that promote the development and function of different memory cells (e.g., *T-bet (Tbx21), Blimp1 (Prdm1), Stat4* and *Eomes*) were upregulated in the tumors of the C215Fab-SEA-treated groups over the course of the study. Furthermore, the analysis of our gene expression data revealed that tumors treated with C215Fab-SEA were also enriched with multiple T-cell memory-associated genes over the course of the study, including *Sell, Il7r, Itgb2 (CD11a), Itga1 (Cd49a), Cd27, Irf4, Irf7, Bcl6b* and the most prominent, *Il12rb2*, which is essential for T-cell memory development (Fig. 7B) [54–56]. The FC results further support the gene data, showing increased frequencies of non-Vβ3 CD8 T cells expressing CD39 (upregulated by antigen-driven activated CD8^+^ T cells)[57] and *Il7r* (Cd127; a marker for memory precursor cells)[58, 59] in both TDLNs and tumors treated with C215Fab-SEA (Fig. 7C, D).

Taken together, these data demonstrate that C215Fab-SEA triggers a strong TCR Vβ3 T-cell response upon re-engagement in the TME, leading to an effective bystander effect by epitope spreading that drives the increased TCR diversity and clonal expansions of non-Vβ3 T cells in the TME. This activation, differentiation and expansion of T cells may give rise to the development of Ag-specific memory T cells and the induction of a long-term memory response against the tumor.

## Discussion

Long-term benefits from cancer immunotherapy, such as ICB therapy, require the induction of immunologic memory through T-cell activation [60]. Many solid tumors lack T-cell infiltration and tumor-specific T-cell activation, which limits the effects of immunotherapy [61]. Our study provides new data demonstrating the dynamics of T-cell activation and overall immunostimulation leading to long-term antitumor immunity following repeated TTS treatments as monotherapy or in combination with ICB agents. We have tested TTS efficacy in the murine B16F10 melanoma model, that is poorly infiltrated by immune cells. Our results demonstrated the capacity of TTS treatment to transform the B16F10 “cold” tumor microenvironment in vivo leading to enhanced T cell infiltration into tumors and prolonged survival. Tumor therapy-productive activation of T cells requires long term activity and pronounced tumor “in situ” effects. We therefore used the MC38 murine tumor model to explore the mechanisms by which TTS alone or in combination with ICB mAbs, leads to a long-term antitumor immune response.

Here, we show that C215Fab-SEA treatment in the MC38-hEpCAM mouse tumor model inhibited tumor growth, prolonged survival, and even resulted in complete tumor clearance following three cycles of treatment. These effects were further enhanced when TTS was combined with ICB (PD-(L)1 inhibitors), resulting in higher rates of tumor-free mice. Cured tumor-free mice were resistant to a second challenge with MC38-hEpCAM and even the parental MC38 cell line 100 days after inoculation. Importantly, although all tumor-free mice were resistant to rechallenge with MC38 colon tumors, they remained susceptible to the newly introduced E0771 breast tumor, indicating tumor-specific long-term immunity. In addition, we showed that T-cell transfer from tumor-free mice to naïve mice also protected against a primary MC38 tumor challenge, which highlights the vital role of T cells in the antitumor memory response induced by TTS treatment.

It was previously shown that SEA selectively activates murine Vβ3 T cells [10]. We found that these activated T cells expanded in the TDLNs over time following TTS treatment. However, while Vβ3 CD4^+^ T cells were found at high frequencies over four treatment cycles, the Vβ3 CD8^+^ T-cell frequencies decreased to baseline levels upon the completion of C2, suggesting that Vβ3 CD8^+^ T cells left the TDLNs to migrate to the tumors. Indeed, the frequencies of Vβ3 CD4^+^ and mainly CD8^+^ T cells increased in the tumors. Interestingly, the number of CD8^+^ Vβ3 T cells decreased in the tumors following C4, while CD4^+^ T-cell numbers remained stable, possibly due to cell death of the CD8^+^ Vβ3 T cells in the TME following serial activation and tumor engagement. This overactivation of T-cell death was reported for CD3-engager bispecific antibodies that were shown to induce apoptotic depletion of antigen-specific T cells in the tumor area due to chronic stimulation [62, 63]. However, unlike CD3 bispecific antibodies, TTSs activate only a small fraction of T cells. While overstimulation by CD3 bispecific antibodies might deplete all tumor-resident T cells, we show here that CD8^+^ T-cell reduction following repeated TTS injections is limited to Vβ3 TTS-reactive T cells. This difference between TTSs and CD3 bispecific antibodies might explain the inability of CD3 bispecific antibodies to install protective memory [62]. The long-term memory antitumor effect induced by TTSs can be attributed to the increased frequencies of non-Vβ3 CD8^+^ T cells following a few cycles of TTS treatment. The increased non-Vβ3 CD8^+^ T-cell frequencies correlated with increased CCR7 expression on these cells, which indicates a strong T-cell influx into the TDLNs. Similarly, the numbers of non-Vβ3 T cells in tumors also increased following TTS treatment, with significant CD8^+^ T-cell accumulation over the study period, with the highest levels after C4. This T-cell redistribution in the TDLNs and the TME might be driven by the regulation of associated chemokines and integrins expressed in the TME following TTS treatment, as demonstrated by gene expression analysis.

We unexpectedly observed a significant reduction in the number of intratumoral Tregs following TTS treatment. This finding was confirmed by FC, NanoString and IHC analyses. Our results contrast with those of an earlier study that showed an increase in Treg numbers following C215Fab-SEA treatment of B16F10 melanoma lung metastases [12]. The difference in results might be due to the treatment schedule that we employed, which more closely mimics the clinical regimen, utilizing several cycles of 4 daily iv injections repeated over 3 weeks. In our study, a profound reduction in Treg numbers was detected by FC analysis after the second, third and fourth cycles of treatment. This decrease in Treg numbers might be due to the significant downregulation of chemokines that are associated with Treg tumor infiltration [34]. Notably, treatment with CD3 bispecific engagers was shown to be limited by the influx of suppressive Treg populations [5, 64]. Hence, the ability of TTS treatment to decrease Treg numbers provides another mechanism that differentiates it from other T-cell engaging treatments and thus may be preferred for therapeutic combination strategies. In addition to the reduction in Tregs, we also detected an increase in the ratio of M1 to M2 TAMs, another marker of a more immune-favorable TME. Unlike TTS therapy, other T-cell-based therapies, such as anti-PD-1/PD-L1 and bispecific CD3 T-cell engager therapies, have been shown to initiate activity in a TME that includes only functional T cells and are less effective in immunosuppressive TMEs [64, 65]. Since TTSs activate T cells outside of the immunosuppressive TME and stimulate T-cell migration into tumors, this treatment can aid in overcoming the suppressive environment, allowing T-cell-based therapies to become more effective in the treatment of solid tumors.

Herein, we have shown for the first time that TTS treatment can be considered an in situ vaccination, an approach that exploits the TAAs available at a tumor site to induce a TAA‐specific adaptive immune response [66, 67]. Following a few cycles of C215Fab-SEA treatment, a strong inflammatory response is provoked by the cytotoxic effect of activated Vβ3 CD8^+^ T cells, releasing tumor (neo)antigens and recruiting non-Vβ3 T cells to the TDLNs, where these cells are primed by APCs that cross-present the released tumor (neo)antigens. We have found that this cross-presentation is mediated by DCs and macrophages, which are initially engaged in Ag uptake in the TME and then migrate into the TDLNs to prime T cells. Antigen spreading is followed by the migration and infiltration of non-Vβ3 T cells into the TME, where they elicit effector functions, further enhancing antitumor responses. This was confirmed by TCR analysis, which showed an increase in the diversity and clonality of non-Vβ3 TCR clones and an upregulation of T-cell memory-associated genes in the TME after treatment. Many genes known to be related to T-cell activation were found to be differentially regulated following C215Fab-SEA treatment, including costimulatory/inhibitory receptor genes, effector function-related genes, and IFN pathway genes. Chronic antigen exposure is expected to lead to T-cell exhaustion, generally characterized by the expression of inhibitory receptors and a reduced ability to secrete effector cytokines. Our FC and gene analysis data revealed that T-cell dysfunction and exhaustion in response to repeated C215Fab-SEA treatments can be reduced by combining C215Fab-SEA with an ICB agent.

Notably, while the combination of C215Fab-SEA with anti-PD-1 treatment showed a synergistic effect in tumor growth inhibition, overall survival and tumor free rates, the superiority of the combination therapy over C215Fab-SEA monotherapy was less evident in the immune profiling results. However, our data shows that the combination therapy induced early increases in T-cell abundance and function in the TME, allowing more intense immune stimulation by C215Fab-SEA and at earlier treatment stages, which can lead to a more profound antitumor response and to a synergistic anti-cancer effect seen in our mouse tumor models.

Overall, according to our data, TTSs induce T-cell migration and tumor infiltration, improve CTL function, reduce the number of Tregs and increase the number of M1 macrophages, converting the immunosuppressive TME into a proinflammatory state. Repeated TTS treatments lead to epitope spreading and to the induction of a T-cell-dependent long-term memory response against the tumor. This unique mechanism of action of TTSs differentiates them from other T-cell engagers and may offer a novel approach to improve immunotherapy efficacy. Moreover, the combination of a TTS with an ICB agent leads to a more profound antitumor effect in vivo, thereby raising hopes for higher response rates in solid cancer patients. The TTS mechanism is currently being investigated in pre-clinical models that reflect the “cold” tumor phenotype. In addition, TTS anti-tumor activity is now being evaluated in orthotopic metastatic models, as well as in combination with CAR-T therapy and other therapeutic approaches. The 5T4-targeted TTS naptumomab estafenatox (NAP) is currently being evaluated in clinical studies in combination with durvalumab [NCT03983954] and docetaxel [NCT04880863].

## Supplementary Information

**Additional file 1:** **Figure S1.** Representative flow cytometry gating strategy for the identification of immune cells. (A) T-cell subsets: CD90.2 + (Thy1.2 +) cells were gated out of single live CD45 + cells. T-cell subsets were further gated according to CD4 and CD8 expression and Vβ3 TCR. Regulatory T cells were gated out from the total CD4 + T cells and evaluated according to CD25 and Foxp3 expression (data not shown). The expression levels of the markers CD137, CD39, CD127, CD103 and CCR7 were determined during the study, and cells were gated according to their matched isotype binding. (B) Myeloid subsets: Single live CD45 + cells were gated according to the expression of CD11b and CD11c lineage markers. DCs were excluded by CD11c + and CD11b- status and were further analyzed for their expression levels of CD103, CD86, MHCII and CCR7; for macrophages and TAMs, CD11b + F4/80 + cells were further analyzed for CD206, CD86, MHCII and CCR7. Marker expression levels were determined according to their matched isotype binding.

**Additional file 2:** **Figure S2.** The combination of C215Fab-SEA and anti-PD-1 significantly inhibited tumor growth, increased survival and induced a protective immune response against tumor rechallenge in MC38 tumor-bearing mice. (A) Schematic illustrating the dosing regimens for mice bearing MC38 tumors. Mice were subcutaneously (s.c.) injected with 5X10^5^ MC38-hEpCAM tumor cells and randomized on Day 5 (≈50 mm^3^ mean tumor volume per group) into treatments of C215Fab-SEA (20 μg/mouse; i.v.), anti-PD-1 mAb (50 μg/mouse; i.p.) or combined therapy. (B) Mean tumor volume (± SEM) of at least 8 mice/group. At Day 19- two-way ANOVA. ***p < 0.0001 treatment vs. control. At Day 22- ***p < 0.0001 combination vs. C215Fab-SEA or anti-PD-1 alone. TGI on Day 19 = 67% vs. control (C) Kaplan‒Meier overall survival curves of treated groups. Survival data were evaluated for statistical significance with the log-rank Mantel‒Cox test. *p = 0.02, **p = 0.006, ***p = 0.0002. n = 10 per group. Tumor-free (TF) mice were rechallenged on Day 75 (50 days following the last treatment). (D) Mean tumor volume (± SEM) of TF mice and naïve control mice that were challenged with MC38-hEpCAM and MC38-parental tumor cells. MC38-hEpCAM tumor cells (5X10^5^) were injected s.c. into the right flank, and 5X10^5^ MC38 parental tumor cells were injected s.c. into the left flank. While 100% of the naïve mice developed flank tumors on both sides, all the pretreated mice completely rejected the second tumor challenge. All the naïve mice died by Day 35 of the study, whereas 100% of the pretreated mice lived for at least 365 days after rechallenge, with no recurrence of the tumors.

**Additional file 3: Figure S3.** The combination of C215Fab-SEA and anti-PD-L1 induced a protective immune response against tumor rechallenge and induced acquired resistance to MC38-hEpCAM tumors via T-cell transfer to naïve mice. **(A)** Schematic illustrating the dosing regimens for the first challenge of mice with MC38 tumors. Mice were subcutaneously (s.c.) injected with 5X10^5^ MC38-hEpCAM tumor cells and randomized on Day 7 (≈50 mm^3^ mean tumor volume per group) into treatments of C215Fab-SEA (20 μg/mouse; i.p.), anti-PD-L1 mAb (100 μg/mouse; i.p.) or combined therapy. (**B)** Individual tumor growth kinetics of mice from the control and treated groups. TF = Tumor-free. (**C)** One hundred days from the start of the study (50 days following the last treatment), tumor-free mice (TF) from the C215Fab-SEA monotherapy group (n = 2) and combination group (Combo; n = 3) and naïve control mice (n = 5) were challenged with MC38-hEpCAM and MC38 parental tumor cells. MC38-hEpCAM tumor cells (5X10^5^) were injected s.c. into the right flank, and 5X10^5^ MC38 parental tumor cells were injected s.c. into the left flank. (**D)** The mean tumor volume kinetics of MC38-hEpCAM (right) and MC38-parental (left) tumors in naïve and TF mice. All the pretreated mice completely rejected the second tumor challenge, whereas 100% of the naïve mice developed flank tumors on both sides. (**E)** On Day 150 of the first tumor challenge study, T cells were isolated from the spleens of three TF mice (mice showing resistance to the second challenge of MC38 tumors) and from untreated naïve mice. A total of 5X10^6^ T cells from the TF or untreated donor mice were adoptively transferred into naïve host mice; 3 days later, the untreated-transferred (ACT naïve; n = 6) and TF-transferred mice (ACT memory; n = 13) were inoculated s.c. with 5X10^5^ MC38-hEpCAM cells, and tumor growth was monitored. **(F)** While 100% of the untreated transferred mice (ACT naïve; n = 6) developed tumors and died by Day 21, 11 out of 13 TF-transferred mice (ACT memory) were resistant to MC38 tumors (left) and showed prolonged survival (right).

**Additional file 4:** **Figure S4.** The combination of the TTS and anti-PD-1 significantly prolonged the survival of tumor-bearing mice and increased T-cell infiltration into lung metastases. **(A)** Schematic illustrating the dosing regimens for mice injected intravenously (i.v.) with B16F10 tumor cells. Mice were injected i.v. with 125,000 B16-hEpCAM tumor cells on Day 0 and randomized for C215Fab-SEA treatment (0.5 µg/mouse; i.v.), anti-PD-1 mAb treatment (200 µg/mouse; i.p.) or combined therapy. (**B)** Kaplan‒Meier overall survival curves of the treated groups as described in Panel A. Survival data were monitored up to Day 90 after tumor inoculation and were evaluated for statistical significance using the log-rank Mantel‒Cox test. The combination (Combo) of C215Fab-SEA with anti-PD1 was significantly more effective than C215Fab-SEA or IgG alone, ***p = 0.0004, n = 10 per group. One mouse from the combination treatment group was tumor-free (TF) at the end of the study. (**C)** Schematic illustrating the dosing regimens of mice injected i.v. with 175,000 B16-hEpCAM tumor cells on Day 0 and randomized on Day 5 for C215Fab-SEA treatment (0.5 µg/mouse; i.v.), anti-PD-1 mAb treatment (200 µg/mouse; i.p.) or combined therapy. On Day 21 postinoculation, the mice were sacrificed, and the lungs were excised for further IHC analysis. **(D)** Frozen sections of lung metastases were analyzed using a Leica DMRX microscope (n = 3/group/cycle). Representative images of IHC double staining. Double labeling: CD3 and granzyme B (GrzB) are marked in green, and CD4 and CD8 are marked in red. Merged markers appear as orange: CD3^+^CD4^+^, CD3^+^CD8^+^ and CD8^+^GrzB^+^. C215Fab-SEA monotherapy led to a massive infiltration of T cells, mostly CD8^+^ T cells, into the tumor and to profound T-cell activation (CD8^+^GrzB^+^). These effects were further enhanced by the combination with anti-PD-1 mAb.

**Additional file 5: Table S1.** Gene sets used to determine cell types in the TME. NanoString pan cancer IO360 list of cell types and the gene signatures that were used to determine the cell abundance in the TME.

## Data Availability

The TTS used in this study, C215Fab-SEA, was manufactured by CRO for NeoTX Therapeutics Ltd. This material is the property of NeoTX Therapeutics Ltd, but TTS compounds with the same structure can be purchased from commercial vendors. This compound is described in a U.S. patent (US11202829B2) assigned to NeoTX Therapeutics Ltd. All data needed to evaluate the conclusions in the paper are available in the main text or the Additional files. Complete flow cytometry analysis datasets, NanoString analysis datasets and TCR sequence datasets are available upon reasonable request.

## Abbreviations

APC: Antigen-presenting cell
CAR-T: Chimeric antigen receptor T cell
CTL: Cytotoxic T lymphocyte
cDC1: Conventional dendritic cell type 1
Fab: Fragment antigen binding
FC: Flowcytometry
ICB: Immune checkpoint blockade
IFN: Interferon
IHC: Immunohistochemistry
MHC: Major histocompatibility complex
NAP: Naptumomab estafenatox
PD-1: Programmed cell death-1 (receptor)
PD-L1: Programmed cell death ligand-1
SEA: Staphylococcal enterotoxin A
SEA/E: Staphylococcal enterotoxin A and E
TAA: Tumor-associated antigens
TAM: Tumor-associated macrophage
TCR: T-cell receptor
TDLN: Tumor draining lymph node
TF: Transcription factor
TME: Tumor microenvironment
Treg: T regulatory cell
TTS: Tumor-targeted superantigen
