# Supplementary figures and images for: Tumor-targeted superantigens produce curative tumor immunity with induction of memory and demonstrated antigen spreading
Source: J Transl Med. 2023 Mar 26;21:222. doi: 10.1186/s12967-023-04064-z (PMC10041807; doi:10.1186/s12967-023-04064-z)

Figure S1

A

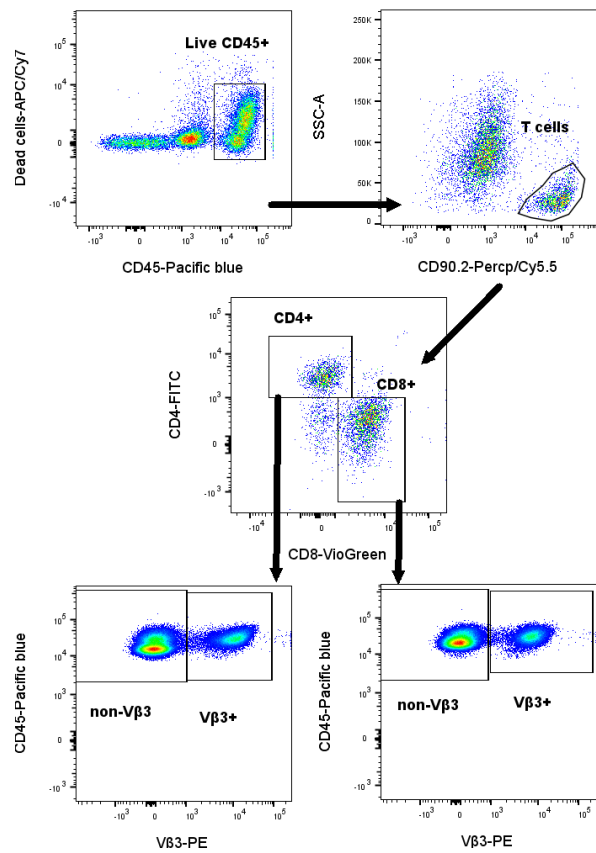

B

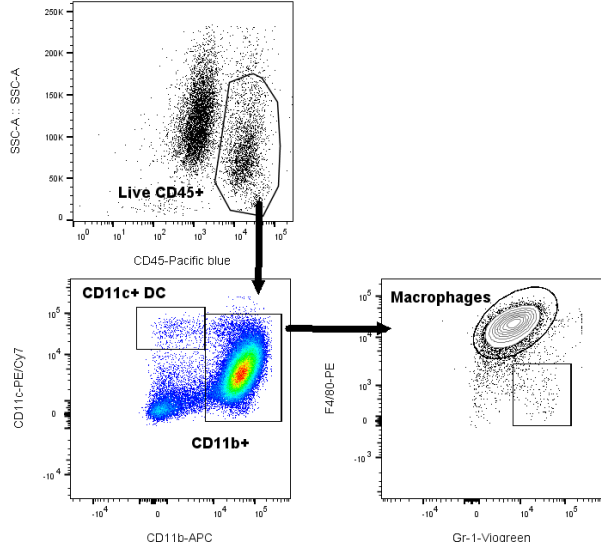

Supplement: Supplementary file 1 — Additional file 1: Figure S1. Representative flow cytometry gating strategy for the identification of immune cells. (A) T-cell subsets: CD90.2 + (Thy1.2 +) cells were gated out of single live CD45 + cells. T-cell subsets were further gated according to CD4 and CD8 expression and Vβ3 TCR. Regulatory T cells were gated out from the total CD4 + T cells and evaluated according to CD25 and Foxp3 expression (data not shown). The expression levels of the markers CD137, CD39, CD127, CD103 and CCR7 were determined during the study, and cells were gated according to their matched isotype binding. (B) Myeloid subsets: Single live CD45 + cells were gated according to the expression of CD11b and CD11c lineage markers. DCs were excluded by CD11c + and CD11b- status and were further analyzed for their expression levels of CD103, CD86, MHCII and CCR7; for macrophages and TAMs, CD11b + F4/80 + cells were further analyzed for CD206, CD86, MHCII and CCR7. Marker expression levels were determined according to their matched isotype binding. [file 12967_2023_4064_MOESM1_ESM.pdf]

Figure S2

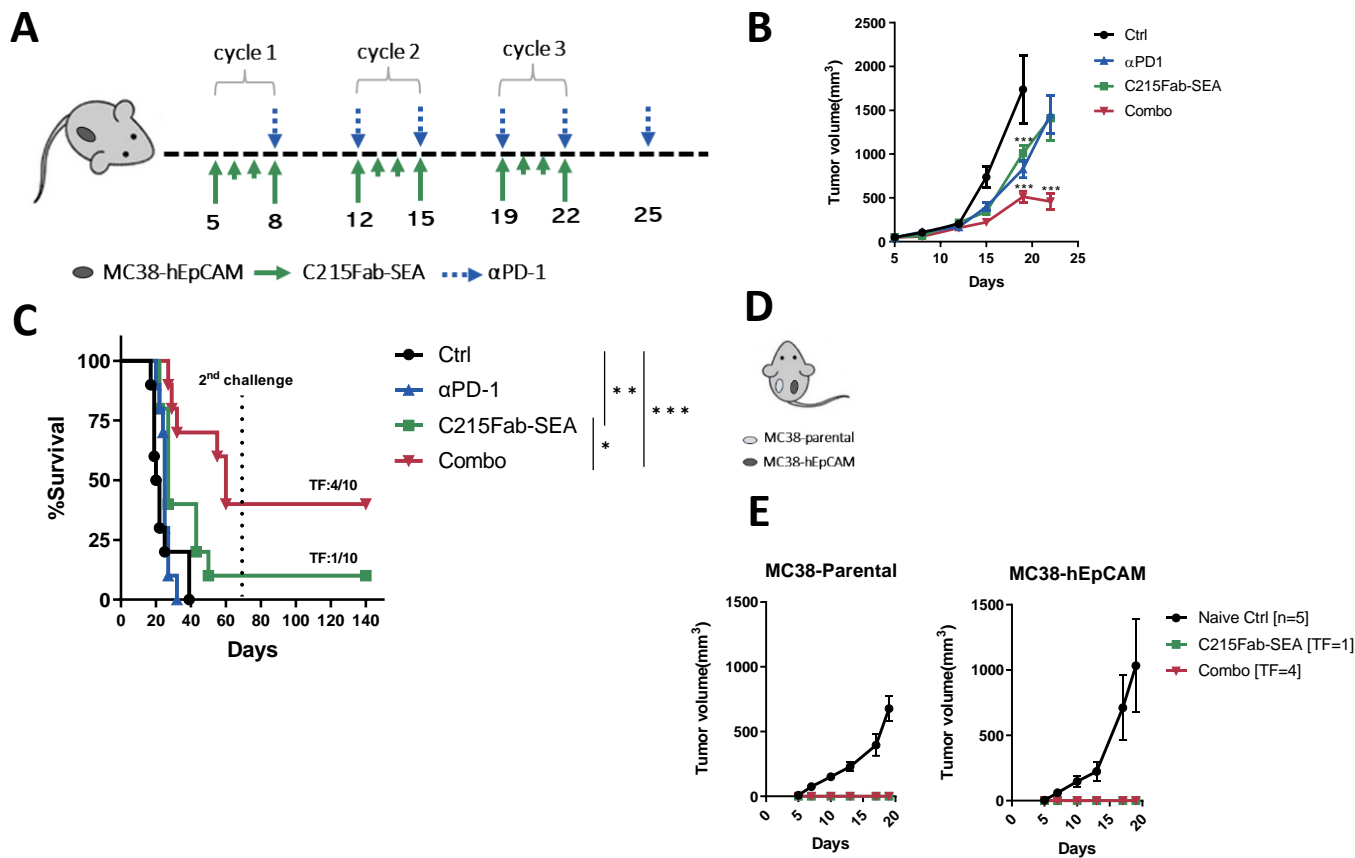

Supplement: Supplementary file 2 — Additional file 2: Figure S2. The combination of C215Fab-SEA and anti-PD-1 significantly inhibited tumor growth, increased survival and induced a protective immune response against tumor rechallenge in MC38 tumor-bearing mice. (A) Schematic illustrating the dosing regimens for mice bearing MC38 tumors. Mice were subcutaneously (s.c.) injected with 5X105 MC38-hEpCAM tumor cells and randomized on Day 5 (≈50 mm3 mean tumor volume per group) into treatments of C215Fab-SEA (20 μg/mouse; i.v.), anti-PD-1 mAb (50 μg/mouse; i.p.) or combined therapy. (B) Mean tumor volume (± SEM) of at least 8 mice/group. At Day 19- two-way ANOVA. ***p < 0.0001 treatment vs. control. At Day 22- ***p < 0.0001 combination vs. C215Fab-SEA or anti-PD-1 alone. TGI on Day 19 = 67% vs. control (C) Kaplan‒Meier overall survival curves of treated groups. Survival data were evaluated for statistical significance with the log-rank Mantel‒Cox test. *p = 0.02, **p = 0.006, ***p = 0.0002. n = 10 per group. Tumor-free (TF) mice were rechallenged on Day 75 (50 days following the last treatment). (D) Mean tumor volume (± SEM) of TF mice and naïve control mice that were challenged with MC38-hEpCAM and MC38-parental tumor cells. MC38-hEpCAM tumor cells (5X105) were injected s.c. into the right flank, and 5X105 MC38 parental tumor cells were injected s.c. into the left flank. While 100% of the naïve mice developed flank tumors on both sides, all the pretreated mice completely rejected the second tumor challenge. All the naïve mice died by Day 35 of the study, whereas 100% of the pretreated mice lived for at least 365 days after rechallenge, with no recurrence of the tumors. [file 12967_2023_4064_MOESM2_ESM.pdf]

Figure S3

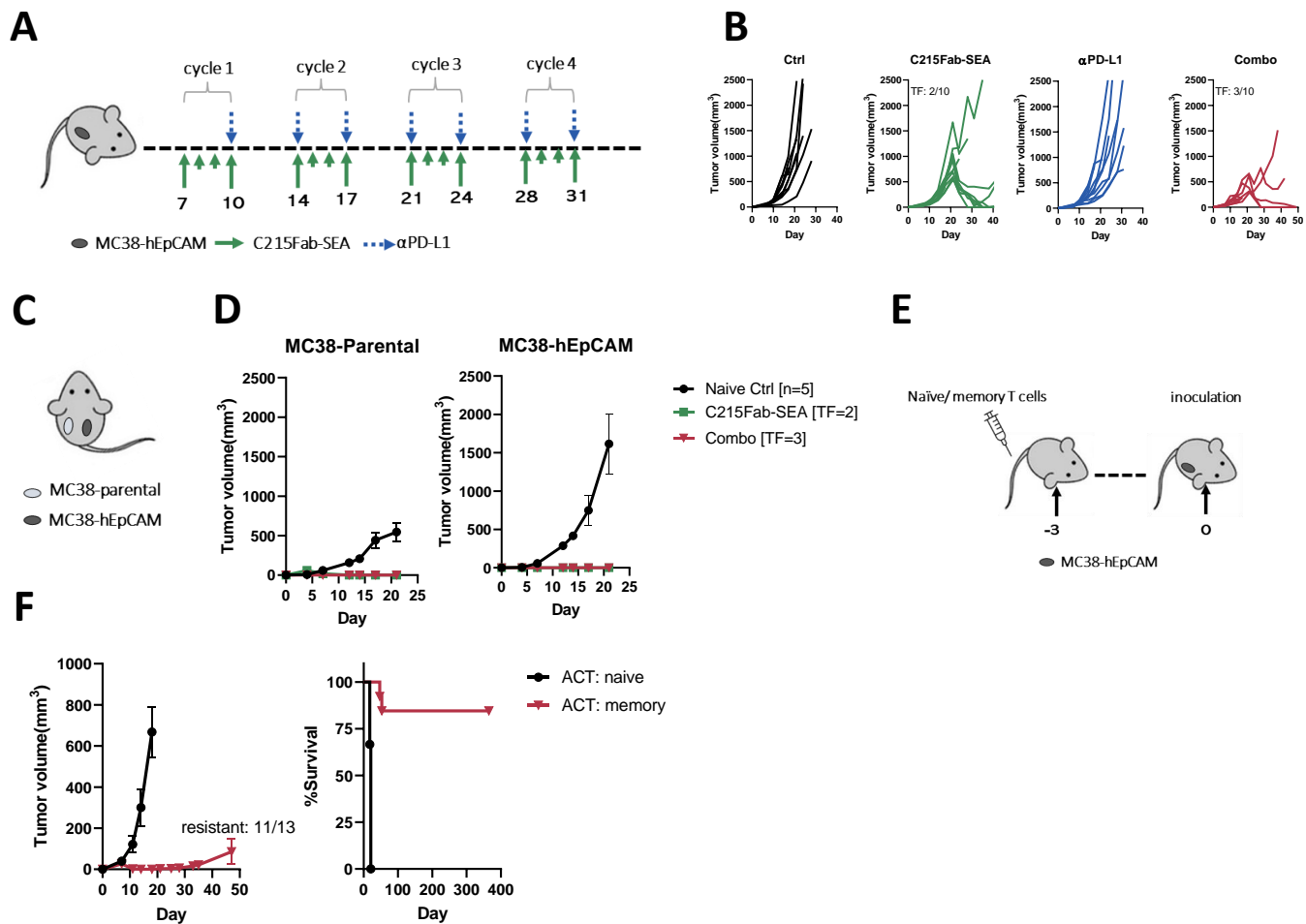

Supplement: Supplementary file 3 — Additional file 3: Figure S3. The combination of C215Fab-SEA and anti-PD-L1 induced a protective immune response against tumor rechallenge and induced acquired resistance to MC38-hEpCAM tumors via T-cell transfer to naïve mice. (A) Schematic illustrating the dosing regimens for the first challenge of mice with MC38 tumors. Mice were subcutaneously (s.c.) injected with 5X105 MC38-hEpCAM tumor cells and randomized on Day 7 (≈50 mm3 mean tumor volume per group) into treatments of C215Fab-SEA (20 μg/mouse; i.p.), anti-PD-L1 mAb (100 μg/mouse; i.p.) or combined therapy. (B) Individual tumor growth kinetics of mice from the control and treated groups. TF = Tumor-free. (C) One hundred days from the start of the study (50 days following the last treatment), tumor-free mice (TF) from the C215Fab-SEA monotherapy group (n = 2) and combination group (Combo; n = 3) and naïve control mice (n = 5) were challenged with MC38-hEpCAM and MC38 parental tumor cells. MC38-hEpCAM tumor cells (5X105) were injected s.c. into the right flank, and 5X105 MC38 parental tumor cells were injected s.c. into the left flank. (D) The mean tumor volume kinetics of MC38-hEpCAM (right) and MC38-parental (left) tumors in naïve and TF mice. All the pretreated mice completely rejected the second tumor challenge, whereas 100% of the naïve mice developed flank tumors on both sides. (E) On Day 150 of the first tumor challenge study, T cells were isolated from the spleens of three TF mice (mice showing resistance to the second challenge of MC38 tumors) and from untreated naïve mice. A total of 5X106 T cells from the TF or untreated donor mice were adoptively transferred into naïve host mice; 3 days later, the untreated-transferred (ACT naïve; n = 6) and TF-transferred mice (ACT memory; n = 13) were inoculated s.c. with 5X105 MC38-hEpCAM cells, and tumor growth was monitored. (F) While 100% of the untreated transferred mice (ACT naïve; n = 6) developed tumors and died by Day 21, 11 out of 13 TF-transferred mice [file 12967_2023_4064_MOESM3_ESM.pdf]

Figure S4

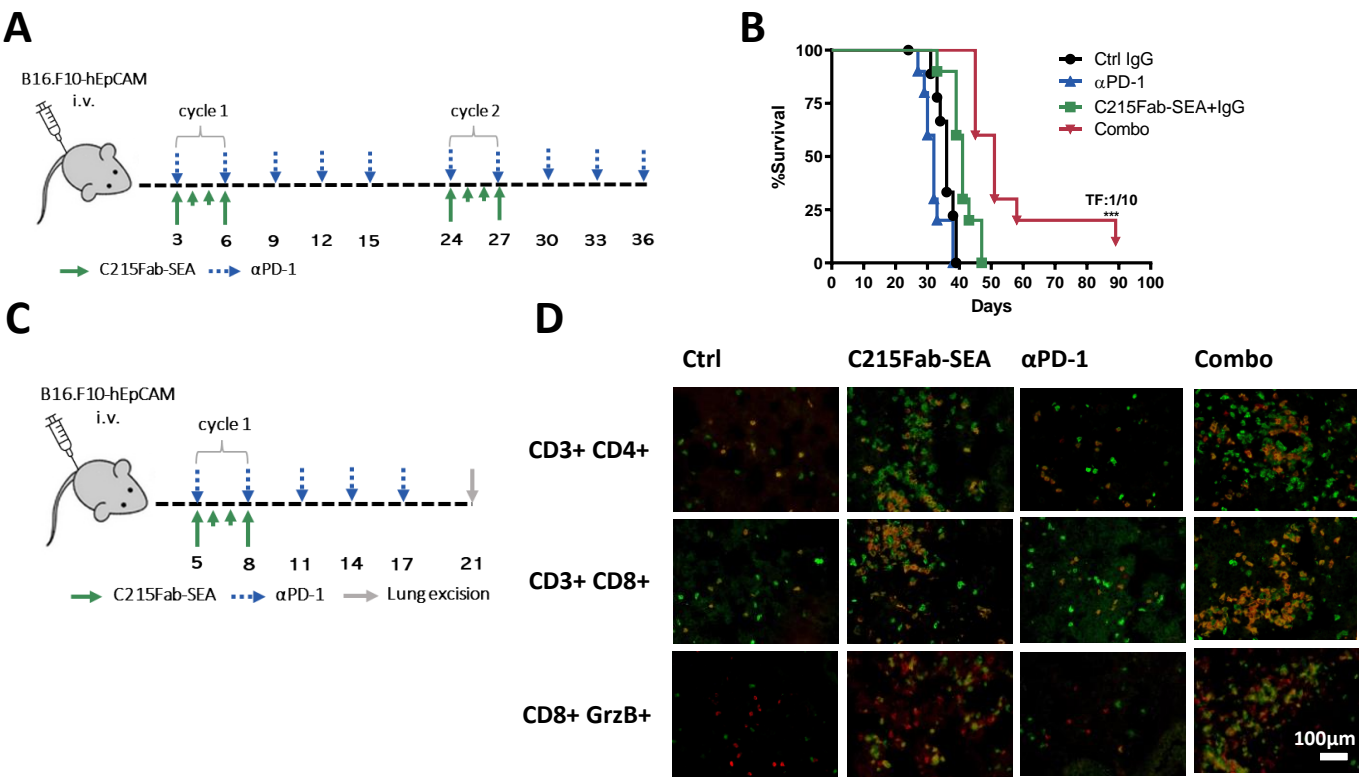

Supplement: Supplementary file 4 — Additional file 4: Figure S4. The combination of the TTS and anti-PD-1 significantly prolonged the survival of tumor-bearing mice and increased T-cell infiltration into lung metastases. (A) Schematic illustrating the dosing regimens for mice injected intravenously (i.v.) with B16F10 tumor cells. Mice were injected i.v. with 125,000 B16-hEpCAM tumor cells on Day 0 and randomized for C215Fab-SEA treatment (0.5 µg/mouse; i.v.), anti-PD-1 mAb treatment (200 µg/mouse; i.p.) or combined therapy. (B) Kaplan‒Meier overall survival curves of the treated groups as described in Panel A. Survival data were monitored up to Day 90 after tumor inoculation and were evaluated for statistical significance using the log-rank Mantel‒Cox test. The combination (Combo) of C215Fab-SEA with anti-PD1 was significantly more effective than C215Fab-SEA or IgG alone, ***p = 0.0004, n = 10 per group. One mouse from the combination treatment group was tumor-free (TF) at the end of the study. (C) Schematic illustrating the dosing regimens of mice injected i.v. with 175,000 B16-hEpCAM tumor cells on Day 0 and randomized on Day 5 for C215Fab-SEA treatment (0.5 µg/mouse; i.v.), anti-PD-1 mAb treatment (200 µg/mouse; i.p.) or combined therapy. On Day 21 postinoculation, the mice were sacrificed, and the lungs were excised for further IHC analysis. (D) Frozen sections of lung metastases were analyzed using a Leica DMRX microscope (n = 3/group/cycle). Representative images of IHC double staining. Double labeling: CD3 and granzyme B (GrzB) are marked in green, and CD4 and CD8 are marked in red. Merged markers appear as orange: CD3+CD4+, CD3+CD8+ and CD8+GrzB+. C215Fab-SEA monotherapy led to a massive infiltration of T cells, mostly CD8+ T cells, into the tumor and to profound T-cell activation (CD8+GrzB+). These effects were further enhanced by the combination with anti-PD-1 mAb. [file 12967_2023_4064_MOESM4_ESM.pdf]
