# Supplementary material for: Tumor-targeted superantigens produce curative tumor immunity with induction of memory and demonstrated antigen spreading
Source: J Transl Med. 2023 Mar 26;21:222. doi: 10.1186/s12967-023-04064-z (PMC10041807; doi:10.1186/s12967-023-04064-z)
Supplement: Supplementary file 5 — Additional file 5: Table S1. Gene sets used to determine cell types in the TME. NanoString pan cancer IO360 list of cell types and the gene signatures that were used to determine the cell abundance in the TME. [file 12967_2023_4064_MOESM5_ESM.pdf]

| Cell type | Gene set |
|---|---|
| CD45+ cells | *Ptprc* |
| B cells | *Blk; Cd19; Ms4a1; Tnfrsf17; Pnoc; Spib; Tcl1* |
| T-cells | *Cd3d; Cd3g; Cd3e; Cd6; Sh2d1a; Trat1* |
| CD8 T cells | *Cd8a; Cd8b1* |
| Th1 cells | *Tbx21* |
| Cytotoxic cells | *Ctsw; Gmza; Gmzb; Klrb1; Klrd1; Klrk1; Prf1; Nkg7* |
| Treg | *Foxp3* |
| DC | *Ccl2; Cd209e; Hsd11b1* |
| Exhausted CD8 | *Cd244; Eomes; Lag3; Ptger4* |
| Macrophages | *Cd163; Cd68; Cd84; Ms4a4a* |
| Neutrophils | *Csfr3; Ceacam3; Fcgr4; Fpr1* |
| NK cells | *Ncr1; Xcl1; Kir3dl1; Kir3dl2* |
